# Reinterpreting the results of the LHC with MadAnalysis 5: uncertainties and higher-luminosity estimates

**DOI:** 10.1140/epjc/s10052-020-8076-6

**Published:** 2020-06-13

**Authors:** Jack Y. Araz, Mariana Frank, Benjamin Fuks

**Affiliations:** 1 Concordia University, 7141 Sherbrooke St. West, Montréal, QC H4B 1R6 Canada; 2 Laboratoire de Physique Théorique et Hautes Energies (LPTHE), UMR 7589, Sorbonne Université et CNRS, 4 place Jussieu, 75252 Paris Cedex 05, France; 3 Institut Universitaire de France, 103 boulevard Saint-Michel, 75005 Paris, France

## Abstract

The MadAnalysis 5 framework can be used to assess the potential of various LHC analyses for unraveling any specific new physics signal. We present an extension of the LHC reinterpretation capabilities of the programme allowing for the inclusion of theoretical and systematical uncertainties on the signal in the reinterpretation procedure. We have implemented extra methods dedicated to the extrapolation of the impact of a given analysis to higher luminosities, including various options for the treatment of the errors. As an application, we study three classes of new physics models. We first focus on a simplified model in which the Standard Model is supplemented by a gluino and a neutralino. We show that uncertainties could in particular degrade the bounds by several hundreds of GeV when considering 3000/fb of future LHC data. We next investigate another supersymmetry-inspired simplified model, in which the Standard Model is extended by a first generation squark species and a neutralino. We reach similar conclusions. Finally, we study a class of *s*-channel dark matter setups and compare the expectation for two types of scenarios differing in the details of the implementation of the mediation between the dark and Standard Model sectors.

## Introduction

The discovery of the Higgs boson has accomplished one of the long awaited objectives of the LHC physics programme and confirmed our understanding of the fundamental laws of nature. However, the concrete realisation of the electroweak symmetry breaking mechanism remains unexplained and no evidence for physics beyond the Standard Model (SM), whose existence is motivated by the SM theoretical inconsistencies and limitations, has emerged from data. There are two classes of possible explanations as to why the associated new particles and/or interactions have escaped detection so far. The first one is that the new states are too heavy and/or the new interactions too feeble to be observed with present collider reaches. Alternatively, new particles may be hiding just around the corner, but lie in a specific configuration (like being organised in a compressed spectrum) that renders their discovery challenging. The possible observation of any new phenomena therefore is the foremost goal of the future LHC runs, including in particular the LHC Run 3, to be started in two years, and the high-luminosity operations planned to begin in half a decade.

In order to investigate whether new physics could be present in existing data, several groups have developed and maintained public software dedicated to the reinterpretation of the results at the LHC [1–5]. In practice, these tools rely on predictions detailing how the different signal regions of given LHC analyses are populated to derive the potential of these searches for its observation. However, signal uncertainties are in general ignored by users in this procedure, although they could sometimes lead to incorrect interpretations [6]. With the limits on the masses of any hypothetical particle being pushed to higher and higher scales, the theoretical uncertainties related with the new physics signals can moreover sometimes be quite severe, in particular if the associated scale and Bjorken-*x* value lead to probing the parton densities in a regime in which they are poorly constrained [7].

On the other hand, it would be valuable to get estimates of the capabilities of the future runs of the LHC with respect to a given signal, possibly on the basis of the interpretation of the results of existing analyses of current data. Predictions in which the signal and the background are naively scaled up could hence be useful to obtain an initial guidance on the reach of future collider setups within new physics parameter spaces.

In this paper, we address the above mentioned issues by presenting an extension of the recasting capabilities of the MadAnalysis 5 platform [3, 8] so that signal theoretical and systematics uncertainties could be included in the recasting procedure. Moreover, we show how the reinterpretation results, with uncertainties included, could be correctly extrapolated to different luminosities to get insight on the sensitivity of the future LHC data on given signals.

As an illustration of these new features within concrete cases, we consider several classes of widely used simplified models. We first extract bounds on various model parameters from recent LHC results. Next, we study how those constraints are expected to evolve with the upcoming high-luminosity run of the LHC through a naive rescaling of the signal and background predictions. In practice, we make use of the recasting capabilities of MadAnalysis 5 and pay a special attention to the theoretical uncertainties.

We begin with a simplified model inspired by the Minimal Supersymmetric Standard Model (MSSM) in which the SM is complemented by a gluino and a neutralino, all other superpartners being assumed heavy and decoupled [9, 10]. Such a particle spectrum leads to a signature comprised of jets and missing transverse energy originating from the gluino decays into an invisible neutralino and quarks. We reinterpret the results of corresponding ATLAS searches for the signal in 36 fb$^{-1}$ [11] and 139 fb$^{-1}$ [12] of LHC data. We investigate the impact of the theory errors on the derived bounds at the nominal luminosity of the search, and extrapolate the findings to estimate the outcome of similar searches analysing 300 and 3000 fb$^{-1}$ of LHC data. Secondly, we make use of these recent LHC searches to perform an equivalent exercise in the context of a simplified model in which the SM is extended by a single species of first generation squarks and a neutralino [9, 10]. Such a spectrum also leads to a new physics signature made of jets and missing transverse energy, although the squark colour triplet nature yields a signal featuring a smaller jet multiplicity. As the considered ATLAS study includes a large set of signal regions each dedicated to a different jet multiplicity, it is sensitive to this simplified model that has moreover not been covered the result interpretations performed in the experimental publication.

As a last example, we study the phenomenology of a simplified dark matter model in which a Dirac fermion dark matter candidate couples to the SM via interactions with an *s*-channel spin-1 mediator [13, 14]. This model is known to be reachable via standard LHC monojet and multijet plus missing transverse energy searches for dark matter. We extract up-to-date bounds on the model by reinterpreting the results of the ATLAS search of Ref. [12] that analyses the full Run 2 ATLAS dataset. This search includes signal regions dedicated to both the monojet and the multijet plus missing energy signatures, so that it consists in an excellent probe for dark matter models. We focus on two specific configurations of our generic simplified models in which the mediator couples with the same strength to the dark and SM sectors. In the first case, we consider mediator couplings of a vector nature, whilst in the second case, we focus on axial-vector mediator couplings. We investigate how the bounds evolve with the luminosity for various dark matter and mediator masses and the nature of the new physics couplings.

The rest of this paper is organised as follows. We discuss the details of the recasting capabilities of MadAnalysis 5 in Sect. 2, focusing not only on the new features that have been implemented in the context of this work, but also on how the code should be used for LHC recasting. We then apply it to extracting gluino and neutralino mass limits in Sect. 3 for various luminosities of LHC data. We analyse the squark/neutralino simplified model in Sect. 4 and perform our dark matter analysis in Sect. 5. We summarise our work and conclude in Sect. 6.

## LHC recasting with MadAnalysis 5

The MadAnalysis 5 package [15, 16] is a framework dedicated to new physics phenomenology. Whilst the first aim of the programme was to facilitate the design and the implementation of analyses targeting a given collider signal of physics beyond the Standard Model, and how to unravel it from the background, more recently it has been extended by LHC reinterpretation capabilities [3, 8]. This feature allows the user to derive the sensitivity of the LHC to any collider signal obtained by matching hard-scattering matrix elements with parton showers, based on the ensemble of analyses that have implemented in the MadAnalysis 5 Public Analysis database (PAD) [3].^1^ For each of these analyses, the code simulates the experimental strategies (which includes both the simulation of the detector response and the selection) to predict the number of signal events that should populate the analysis signal regions. It then compares the results with both data and the SM expectation, so that conclusive statements could be drawn. As in all recasting codes relying on the same method [2, 4, 5], the uncertainty on the signal is ignored although it could be relevant [7].

With the release of MadAnalysis 5 version v1.8, the user has now the possibility to deal with various classes of signal uncertainties and to extrapolate any reinterpretation result to higher luminosities. This section documents all these new functionalities. Section 2.1 briefly summarises how to install MadAnalysis 5, get the code running and download a local copy of its public analysis database. Section 2.2 details how the code can be used to reinterpret the results of a specific LHC analysis. A more extensive and longer version of this information on MadAnalysis 5 installation and running procedures can be found in Ref. [8]. Section 2.3 is dedicated to the new methods that have been developed in the context of this work, and which are available from MadAnalysis 5 version v1.8 onwards. We also introduce in this section several new optional features that can be used for the design of the analysis .info files. One such file accompanies each analysis of the database and contains information on the observation and the SM expectation of the different analysis signal regions. In Sect. 2.4, we describe the corresponding modifications of the output format relevant for a recasting run of MadAnalysis 5.

### Prerequisites and installation

MadAnalysis 5 is compatible with most recent Unix-based operating systems, and requires the GNU G++ or CLang compiler, a Python 2.7 installation (or more recent, but not a Python 3 one) and GMake. In order for the recasting functionalities to be enabled, the user must ensure that the SciPy library is present, as it allows for limit computations, and that the Delphes 3 package [17] is locally available within the MadAnalysis 5 installation. The latter, which requires the Root framework [18] and the FastJet programme [19], is internally called by MadAnalysis 5 to deal with the simulation of the response of the LHC detectors and to reconstruct the events. Moreover, reading compressed event files can only be performed if the Zlib library is available.

The latest version of MadAnalysis 5 can be downloaded from LaunchPad,^2^ where it is provided as a tarball named $\mathtt{ma5\_v<xxx>.tgz}$, that contains all MadAnalysis 5 source files ($\mathtt{<xxx>}$ standing for the version number). After unpacking the tarball, the code can be started by issuing in a shell 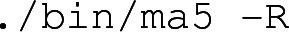 where the -R options enforces the reco mode of MadAnalysis 5, that is relevant for LHC recasting. The programme begins with checking the presence of all mandatory packages and determining which of the optional packages are available. The MadAnalysis 5 command-line interface is then initialised and the user is prompted to type in commands.

In the case where any of the Zlib or Delphes 3 package would not be found by MadAnalysis 5, they can be installed locally by typing, directly in the MadAnalysis 5 interpreter, 
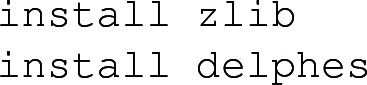
 Whilst Root can in principle be installed similarly, we recommend the user to handle this manually, following the instructions available on the Root website.^3^ Furthermore, all existing and validated recast LHC analyses in the MadAnalysis 5 framework can be locally downloaded by typing in, 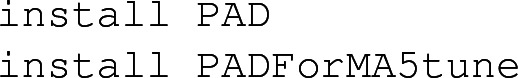 The second command triggers the installation of older implemented analyses, that requires a (now disfavoured) MA5tune version of Delphes 3. The latter can be installed by typing, in the MadAnalysis 5 shell, 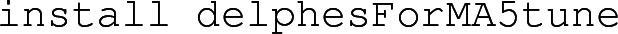


### Recasting LHC analyses with MadAnalysis 5

In this section, we rely on a generic example in which a user aims to estimate the sensitivity of a specific LHC analysis to a given signal with MadAnalysis 5. The analysis consists of one of the analyses available from the PAD and the signal is described by simulated events collected into a file that we call events.hepmc.gz. Such an event file includes the simulation of the considered hard-scattering process matched with parton showers, as well as the hadronisation of the final-state partons present in each of the showered events.

As mentioned above, MadAnalysis 5 has to be started in the reco mode, 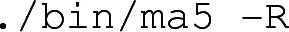 In a first step, the recasting mode of the programme has to be enabled and the event file, physically located at $\texttt {<path-to-events.hepmc.gz>}$ on the user system, has to be imported. This is achieved by issuing the commands 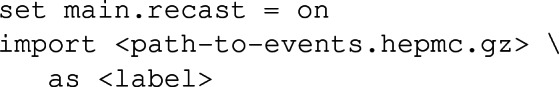 The second command defines a dataset identified by the label $\texttt {<label>}$ that here solely includes the imported sample. Several event files can be imported and collected either under a unique dataset (by using the same $\texttt {<label>}$ for each call to the import command) or split into different datasets (by employing different labels). When studying the signal under consideration, MadAnalysis 5 will run over all defined datasets and imported event files.

In addition, the user can activate the storage of the Root file(s) generated by Delphes 3 by issuing the command, 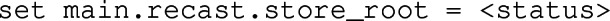 where $\mathtt{<status>}$ can take the True or False value, and directly provide a predefined recasting card (available on the system at $\mathtt{<path-to-a-card>}$), through 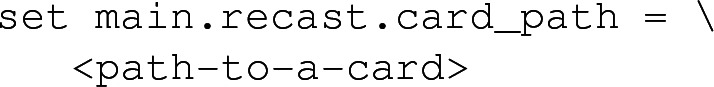 In the case where no card is provided, MadAnalysis 5 creates a consistent new card with one entry for each of the available analyses. Such an entry is of the form
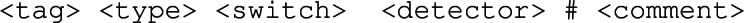
The $\texttt {<tag>}$ label corresponds to the filename of the C++ code associated with the considered analysis (located in the Build/SampleAnalyzer/User/Analyzer subdirectory of the PAD installation in tools/PAD), the $\texttt {<type>}$ label indicates whether the PADForMA5tune (v1.1) or PAD (v1.2) recasting infrastructure should be used and the $\texttt {<switch>}$ tag (to be set to on or off) drives whether the analysis has to be recast. The name of the Delphes 3 card to use (see the Input/Cards subdirectory of the PAD installation) is passed as $\texttt {<detector>}$, and $\texttt {<comment>}$ consists of an optional comment (usually briefly describing the analysis).

The run is finally started by typing in the interpreter, 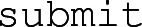 Firstly, MadAnalysis 5 simulates the detector impact on the input events, for each of the necessary Delphes 3 cards according to the analyses that have been switched on in the recasting card. Next, the code derives how the different signal regions are populated by the signal events and finally computes, by means of the CL$_s$ prescription [20], the corresponding exclusion limits, signal region by signal region. This is achieved by a comparison of the results with the information on the SM background and data available from the different info files shipped with the PAD.

The output information is collected into a folder named ANALYSIS_X, where X stands for the next available positive integer (in terms of non-existing directories). On top of basic details about the run itself, this folder contains the recasting results that are located in the ANALYSIS_X/Output folder. The latter includes the CLs_output_summary.dat file that concisely summarises all the results of the run. A more extensive version of these results can be found in the set of subfolders named after the labels of the imported datasets. The CLs_output_summary.dat file contains one line for each signal region of each reinterpreted analysis, and this for each of the datasets under consideration. Each of these lines follows the format
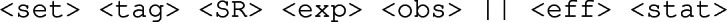


where the $\texttt {<set>}$ and $\texttt {<tag>}$ elements respectively consist in the names of the dataset and analysis relevant for the considered line of the output file. The $\texttt {<SR>}$ entry relates to one of the analysis signal regions, the exact name being the one defined in the analysis C++ source code. The $\texttt {<exp>}$ and $\texttt {<obs>}$ quantities are the expected and observed cross-section values for which the signal modelled by the events stored within the dataset $\texttt {<set>}$ is excluded by the signal region $\texttt {<SR>}$ of the analysis $\texttt {<tag>}$ at the 95% confidence level. In the former case, the code makes use of the SM expectation to predict the number of events populating the signal region $\texttt {<SR>}$, whilst in the latter case, data is used. Finally, the $\texttt {<eff>}$ and $\texttt {<stat>}$ entries respectively refer to the corresponding selection efficiency and the associated statistical error.

The user has the option to specify the cross section corresponding to the investigated signal by issuing, in the MadAnalysis 5 interpreter, 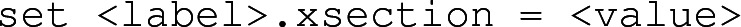 prior to the call to the submit command. Following this syntax, $\texttt {<label>}$ stands for one of the labels of the considered datasets and $\texttt {<value>}$ for the associated cross-section value, in pb. In this case, the confidence level at which the analysed signal is excluded is included in the output summary file (before the double vertical line).

The Output folder additionally contains a specific subfolder for each of the defined datasets. Such a directory contains a file named CLs_output.dat that includes the same information as in the CLs_output_summary.dat file, following the same syntax, but restricted to a specific dataset. A second file encoded into the SAF format [15] and named $\texttt {<label>.saf}$ ($\texttt {<label>}$ being the dataset name) contains general information on the dataset organised according to an XML-like structure. The latter relies on three classes of elements, namely $\texttt {<SampleGlobalInfo>}$, $\texttt {<FileInfo>}$ and $\texttt {<SampleDetailedInfo>}$. The first of these contains global information on the dataset, such as its cross section (xsec), the associated error (xsec_err), the number of events (nev) or the sum of the positive and negative event weights (sum_w and sum_w-). The corresponding entry in the output file would read
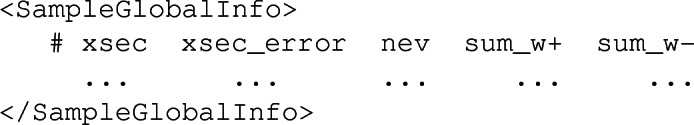
where the numerical values have been omitted for clarity. The $\texttt {<FileInfo>}$ element sequentially provides the paths to the different event files included in the dataset, while detailed information on each file is provided within the $\texttt {<SampleDetailedInfo>}$ XML root element, in a similar manner as for the sample global information (with one line for each file).

Furthermore, the dataset output directory includes a RecoEvents folder dedicated to the storage of Delphes 3 output files (one file for each considered detector parameterisation), provided that the corresponding option has been turned on (see above), as well as one folder for each of the recast analyses. Each of these folders contains one SAF file listing all signal regions implemented in the associated analysis, as well as two subfolders Cutflows and Histograms. The former includes one SAF file for each signal region, and the latter a single file named histos.saf.

A cutflow is organised through XML-like elements, $\texttt {<InitialCounter>}$ and $\texttt {<Counter>}$ being used for the initial number of events and the results of each selection cut respectively. As depicted by the example below, in which all numbers have been omitted for clarity, 
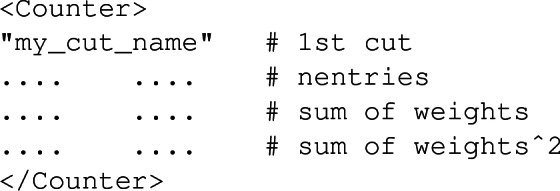
 any of such elements includes a cut name as defined in the analysis C++ file (first line), the number of events passing the cut (second line), the weighted number of events passing the cut (third line) and the sum of the squared weights of all events passing the cut (last line). Moreover, the first (second) column refers to the positively-weighted (negatively-weighted) events only.

Histograms are all collected into the file histos.saf, that is also organised according to an XML-like structure relying on several $\texttt {<Histo>}$ elements. Each of these corresponds to one of the histograms implemented in the analysis. A $\texttt {<Histo>}$ element includes the definition of the histogram (provided within the $\texttt {<Description>}$ element), general statistics (as part of the $\texttt {<Statistics>}$ element) and the histogram data itself (within the $\texttt {<Data>}$ element). The description of a histogram schematically reads 
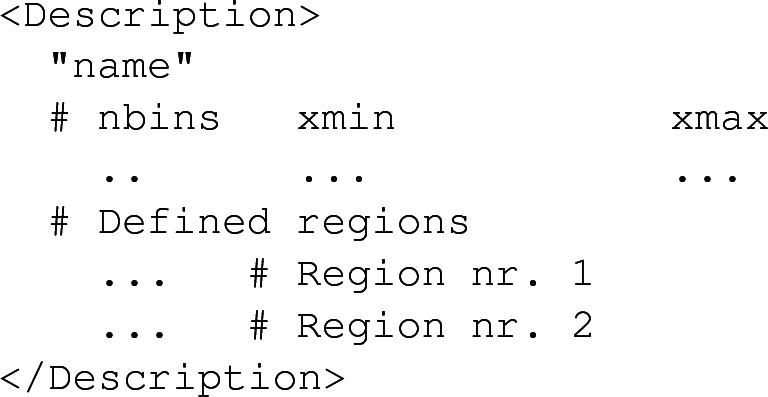
 and is self-explanatory, all numbers having been replaced by dots. This moreover shows that a given histogram can be associated with several signal regions, provided they are indistinguishable at the moment the histogram is filled. Statistics are typically given as
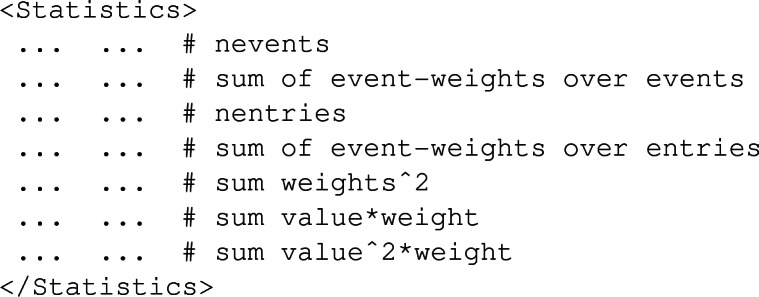
which include information about the number of entries, the weighted number of entries, the variance, *etc.* Moreover, the contributions of the positively-weighted and negatively-weighted events are again split and provided within the first and second column respectively. The values of each bin are finally available from the $\texttt {<Data>}$ element, 
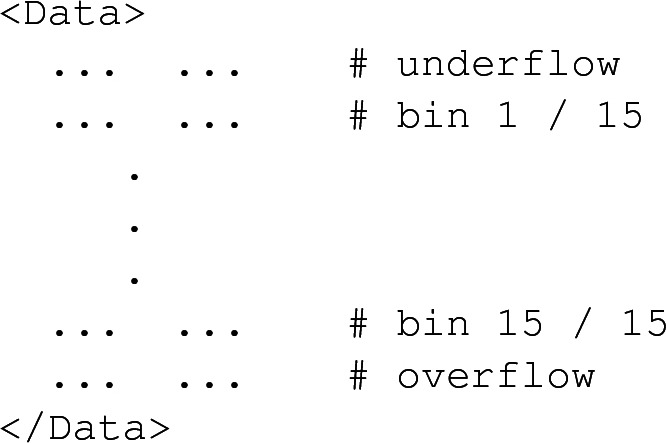
 where all bin values are omitted and the two columns respectively refer to events with positive (first column) and negative (second column) weights. The underflow and overflow bins are also included.

To close this section, we detail below how limits on a given signal are derived by MadAnalysis 5, using the CL$_s$ prescription. The output file generated by the code contains three numbers associated with those limits, the expected and observed cross sections excluded at the 95% confidence level, $\sigma _{95}^\mathrm{exp}$ and $\sigma _{95}^\mathrm{obs}$, as well as the confidence level at which the input signal is excluded. Those numbers are extracted on the basis of the information available from the .info file, shipped with each recast analysis and that contains, for each signal region, the number of expected SM events $n_b$, the associated error $\varDelta n_b$ and the observed number of events populating the signal region $n_\mathrm{obs}$. As said above, starting from the input event file, MadAnalysis 5 simulates the response of the LHC detector, applies the analysis selection, and estimates how the different signal regions are populated. In this way, for each signal region, the number of signal events $n_s$ is known.

This enables the computation of the background-only and signal-plus-background probabilities $p_\mathrm{b}$ and $p_{\mathrm{b} + \mathrm{s}}$ and to further derive the related CL$_s$ exclusion. In practice, the code considers a number of toy experiments (the default being 100000 that can be changed by issuing, in the MadAnalysis 5 interpreter and before the call to the submit method, 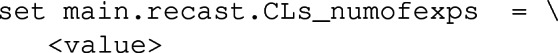 where $\texttt {<value>}$ stands for the desired number of toy experiments. For each toy experiment, the expected number of background events $N_\mathrm{b}$ is randomly chosen assuming that its distribution is Gaussian, with a mean $n_\mathrm{b}$ and a width $\varDelta n_\mathrm{b}$. The corresponding probability density thus reads

1 $$\begin{aligned} f(N_b|n_b,\varDelta n_b) = \frac{\exp \left\{ -\frac{(N_b - n_b)^2}{2\varDelta n_b^2}\right\} }{\varDelta n_b\sqrt{2\pi }}. \end{aligned}$$

Imposing $N_\mathrm{b} \ge 0$, the actual number of background events $\hat{N}_\mathrm{b}$ is randomly generated from the Poisson distribution

2 $$\begin{aligned} f(\hat{N}_b|N_b) = \frac{N^{\hat{N}_b}_be^{-N_b}}{\hat{N}_b!}. \end{aligned}$$

Accounting for the observation of $n_\mathrm{obs}$ events, $p_\mathrm{b}$ is defined as the percentile of score associated with $\hat{N}_\mathrm{b} \le n_\mathrm{obs}$, which consists in the probability for the background to fluctuate as low as $n_\mathrm{obs}$.

The signal-plus-background probability $p_{\mathrm{b} + \mathrm{s}}$ is computed similarly, assuming that the actual number of signal-plus-background events $\hat{N}_\mathrm{b}+\hat{N}_\mathrm{s}$ follows a Poisson distribution of parameter $n_\mathrm{s}+N_\mathrm{b}$ (after imposing this time that $N_\mathrm{b} + n_\mathrm{s}>0$). The resulting CL$_s$ exclusion is then derived as

3 $$\begin{aligned} \mathrm{CL}_s = \mathrm{max}\Big (0, 1-\frac{p_{\mathrm{b}+\mathrm{s}}}{p_\mathrm{b}}\Big ). \end{aligned}$$

and $\sigma _{95}^\mathrm{obs}$ is calculated as above in a case where the number of signal events $n_s$ is kept free. From the (derived) knowledge of the analysis selection efficiencies, MadAnalysis 5 can extract the upper allowed cross section value for which the signal is not excluded, *i.e.* $\sigma _{95}^\mathrm{obs}$. The expected cross section excluded at the 95% confidence level, $\sigma _{95}^\mathrm{exp}$, is obtained by replacing $n_\mathrm{obs}$ by $n_\mathrm{b}$ in the above calculations.

### Including signal uncertainties and extrapolation to higher luminosities

In the procedure described in the previous section, any error on the signal is ignored, both concerning the usual theory uncertainties (scale variations, parton densities) and the systematics, mostly stemming from more experimental aspects. In particular, with the constantly growing mass bounds on hypothetical new particles, the scale entering the relevant hard-scattering processes is larger and larger, so that theoretical errors could start to impact the derived limits in an important and non-negligible manner.

Starting from version v1.8 onwards, MadAnalysis 5 offers the user a way to account for both the theoretical and systematical errors on the signal when a limit calculation is performed. The scale and parton density (PDF) uncertainties can be entered, within the MadAnalysis 5 interpreter, similarly to the cross section associated with a given dataset (see Sect. 2.2),
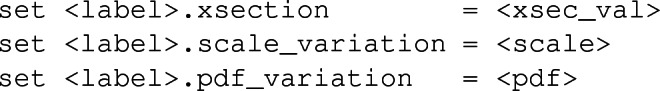
where $\texttt {<label>}$ stands for the label defining the signal dataset. In this case, the signal cross section $\sigma _s$ is provided through the xsection attribute of the dataset, as described in the previous section, while the scale and parton density uncertainties $\varDelta \sigma _\mathrm{scales}$ and $\varDelta \sigma _\mathrm{PDF}$ are given through the scale_variation and pdf_variation attributes. The errors are symmetric with respect to the central value $\sigma _s$, and their value (given by $\texttt {<scale>}$ and $\texttt {<pdf>}$ in the above example) must be inputted as the absolute values of the relative errors on the cross section (*i.e.* as positive floating-point numbers). Asymmetric errors can also be provided, the upper and lower uncertainties being independently fixed by issuing, in the MadAnalysis 5 interpreter,
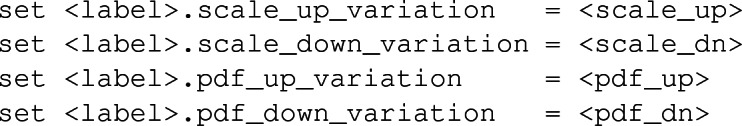
Each error is again provided as a positive floating-point number and refers to the relative error on the cross section, in absolute value. On top of the computation of the confidence level at which the signal is excluded, MadAnalysis 5 additionally calculates the CL$_s$ variation band associated with the scale uncertainties, as well as with the total theory uncertainties where both the scale and PDF contributions to the total error are added linearly. Such a behaviour can however be modified by issuing, in the interpreter 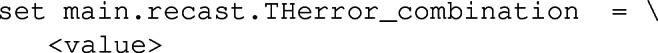 where $\texttt {<value>}$ can be set either to quadratic (the theory errors are added quadratically) or linear (default, the theory errors are added linearly). The CL$_s$ band is then derived by allowing the signal cross section to vary within its error band, deriving the associated spread on $p_\mathrm{{b}+\mathrm {s}}$.

The user can also specify one or more values for the level of systematics on the signal. This is achieved by issuing, in the command line interface, 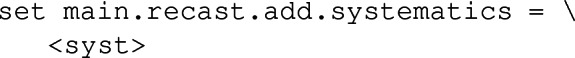 This command can be reissued as many times as needed, MadAnalysis 5 taking care of the limit calculation for each entered value independently. The level of systematics ($\texttt {<syst>}$) has to be given either as a floating-point number lying in the [0, 1] range, or as a pair of floating-point numbers lying in the same interval. In the former case, the error is symmetric with respect to the central value $\sigma _s$, whilst in the latter case, it is asymmetric with the first value being associated with the upper error and the second one with the lower error.

In addition, we have also extended the code so that naive extrapolations for a different luminosity $\mathcal{L}_\mathrm{new}$ could be performed. This is achieved by typing, in the interpreter,
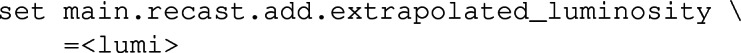
Once again, the user has the possibility to reissue the command several times, so that the extrapolation will be performed for each luminosity $\texttt {<lumi>}$ independently (where the value has to be provided in fb$^{-1}$). Those extrapolations assume that the signal and background selection efficiencies of a given region in a specific analysis are identical to those corresponding to the reference luminosity $\mathcal{L}_0$ initially considered. In this framework, the extrapolated number of background events $n_b^\mathrm{new}$ is related to $n_b$ (the number of background events expected for the reference luminosity $\mathcal{L}_0$) as

4 $$\begin{aligned} n_b^\mathrm{new} = n_b\ \frac{\mathcal {L}_\mathrm{new}}{\mathcal {L}_0}, \end{aligned}$$

that we assume equal to the extrapolated number of observed events,

5 $$\begin{aligned} n_\mathrm{obs}^\mathrm{new} = n_b^\mathrm{new}. \end{aligned}$$

On the other hand, the associated uncertainties, $\varDelta n_b^\mathrm{new}$, are derived from the relation

6 $$\begin{aligned} \varDelta n_b^\mathrm{new} = \varDelta _{b, \mathrm{syst}} \frac{\mathcal{L}_\mathrm{new}}{\mathcal{L}_0} \oplus \varDelta _{b, \mathrm{stat}} \sqrt{\frac{\mathcal{L}_\mathrm{new}}{\mathcal{L}_0}}, \end{aligned}$$

where the statistics and systematics components are added in quadrature. The systematics are extrapolated linearly, whilst the statistical uncertainties assume that the event counts follow a Poisson distribution. Such an extrapolation of the background error requires an access to the details of the background uncertainties. This is however not achievable within the XML info file format dedicated to the transfer of the background and data information to MadAnalysis 5 [3]. We therefore introduce two new XML elements to this format, namely deltanb_stat and deltanb_syst. These offer the user the option to implement his/her info file by either providing a unique combined value for the uncertainties (via the standard deltanb XML element) or by splitting them into their statistical and systematical components (via a joint use of the new deltanb_stat and deltanb_syst XML elements). In this way, a region element could be either implemented according to the old syntax, as in the schematic example below (with all numbers omitted), 
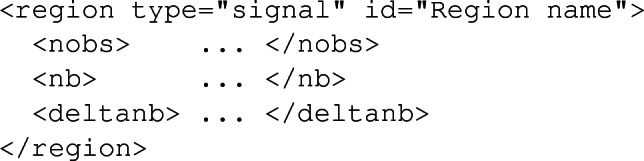
 or following the new syntax, which would then read 
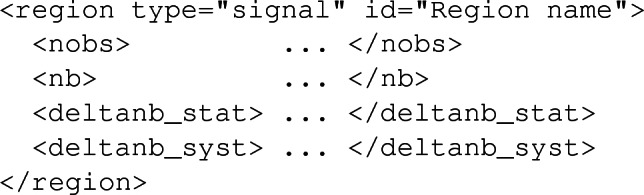
 Whilst the usage of the new syntax is encouraged, this new possibility for embedding the error information strongly depends on how the background uncertainties are provided in the experimental analysis notes. For this reason, as well as for backward-compatibility, MadAnalysis 5 supports both choices. If only a global error is provided, the user can freely choose how to scale the error (linearly or in a Poisson way), by typing in the interpreter, 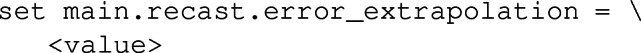 where $\texttt {<value>}$ has to be set either to linear or to sqrt. The user has also the choice to use a single floating-point number for the $\texttt {<value>}$ parameter. In this case, the relative error on the number of background events at the new luminosity, $\varDelta n_b^\mathrm{new}/n_b^\mathrm{new}$, is taken equal to this number. Finally, the user can provide a comma-separated pair of floating-point numbers $\kappa _1$ and $\kappa _2$, as in 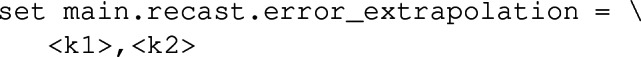 The background error is here defined by

7 $$\begin{aligned} \bigg [\frac{\varDelta n_b^\mathrm{new}}{n_b^\mathrm{new}}\bigg ]^2 = \kappa _1^2 + \frac{\kappa _2^2}{n_b}, \end{aligned}$$

where the two values provided by the user respectively control the systematical component of the uncertainties ($\texttt {<k1>}$, $\kappa _1$) and the statistical one ($\texttt {<k2>}$, $\kappa _2$). Finally, all extrapolations are based on expectations and not on observations, so that $n_\mathrm{obs}$ will be effectively replaced by the corresponding SM expectation $n_\mathrm{b}$.

### Output format

MadAnalysis 5 propagates the information on the impact of the uncertainties all through the output file, which is then written in a format slightly extending the one presented in Sect. 2.2. Starting with the summary file CLs_output_summary.dat, each line (corresponding to a given signal region of a given analysis) is now followed by information schematically written as
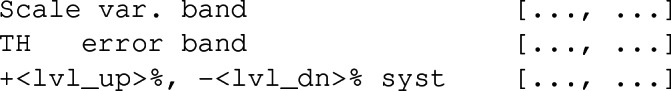
The uncertainties on the exclusion stemming from scale variations are given in the first line, which is trivially omitted if the corresponding information on the signal cross section is not provided by the user. In the second line, MadAnalysis 5 adds either quadratically or linearly (according to the choice of the user) all theory errors, such a line being written only if at least one source of theory uncertainties is provided by the user. Finally, if the user inputted one or more options for the level of systematics, MadAnalysis 5 computes the band resulting from the combination of all errors and writes it into the output file (one line for each choice of level of systematics). In the above snippet, the user fixed an asymmetric level of systematics (for the sake of the example) indicated by the $\texttt {<lvl\_up>}$ and $\texttt {<lvl\_dn>}$ tags.

In cases where the band would have a vanishing size, the uncertainty information is not written to the output file. This could be due either to negligibly small uncertainties, to the fact that for the considered region, the signal is excluded regardless the level of systematics (at the 100% confidence level), or to the region not targeting the signal at all (the corresponding selection efficiency being close to zero).

The CLs_output.dat dataset-specific files present in the output subdirectory associated with each imported dataset all contain similar modifications. In case of extrapolations to different luminosities, copies of this file named $\texttt {CLs\_output\_lumi\_<lumi>.dat}$ are provided for each desired luminosity $\texttt {<lumi>}$.

## Gluino and neutralino mass limits

**Fig. 1:**
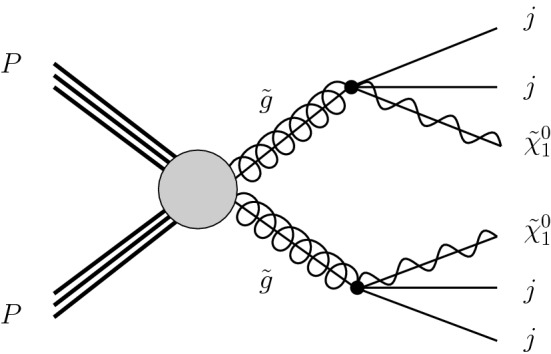
Generic Feynman diagram associated with the production and decay of a pair of gluinos in the considered MSSM-inspired gluino simplified model. The figure has been produced with the help of the JaxoDraw package [21]

To illustrate the usage of the new functionalities of MadAnalysis 5 introduced in the previous section, we perform several calculations in the context of a simplified model inspired by the MSSM. In this framework, all superpartners are heavy and decoupled, with the exception of the gluino $\tilde{g}$ and the lightest neutralino $\tilde{\chi }_1^0$, taken to be bino-like. Any given benchmark is thus defined by two parameters, namely the gluino and the neutralino masses $m_{\tilde{g}}$ and $m_{\tilde{\chi }_1^0}$. Such a new physics setup can typically manifest itself at the LHC through a signature made of a large hadronic activity and missing transverse energy. As shown by the schematic Feynman diagram of Fig. 1, such a signature originates from the production of a pair of gluinos, each of them promptly decaying into two jets and a neutralino (via virtual squark contributions).

We study the sensitivity of the LHC and its higher-luminosity upgrades to this signal by analysing state-of-the-art Monte Carlo simulations achieved by means of the MG5_aMC framework (version 2.6.6) [22], using the MSSM-NLO model implementation developed in Ref. [7]. Hard-scattering matrix elements are generated at the next-to-leading-order (NLO) accuracy in QCD and convoluted with the NLO set of NNPDF 3.0 parton densities [23], as provided by the LHAPDF interface [24]. The gluino leading-order (LO) decays are handled with the MadSpin [25] and MadWidth [26] packages. The resulting NLO matrix elements are then matched with Pythia parton showers and hadronisation (version 8.240) [27], following the MC@NLO method [28]. Our predictions include theoretical uncertainties stemming from the independent variations of the renormalisation and factorisation scales by a factor of two up and down relatively to the central scale, taken as half the sum of the transverse masses of the final-state particles, as well as from the parton densities extracted following the recommendations of Ref. [29].

**Fig. 2:**
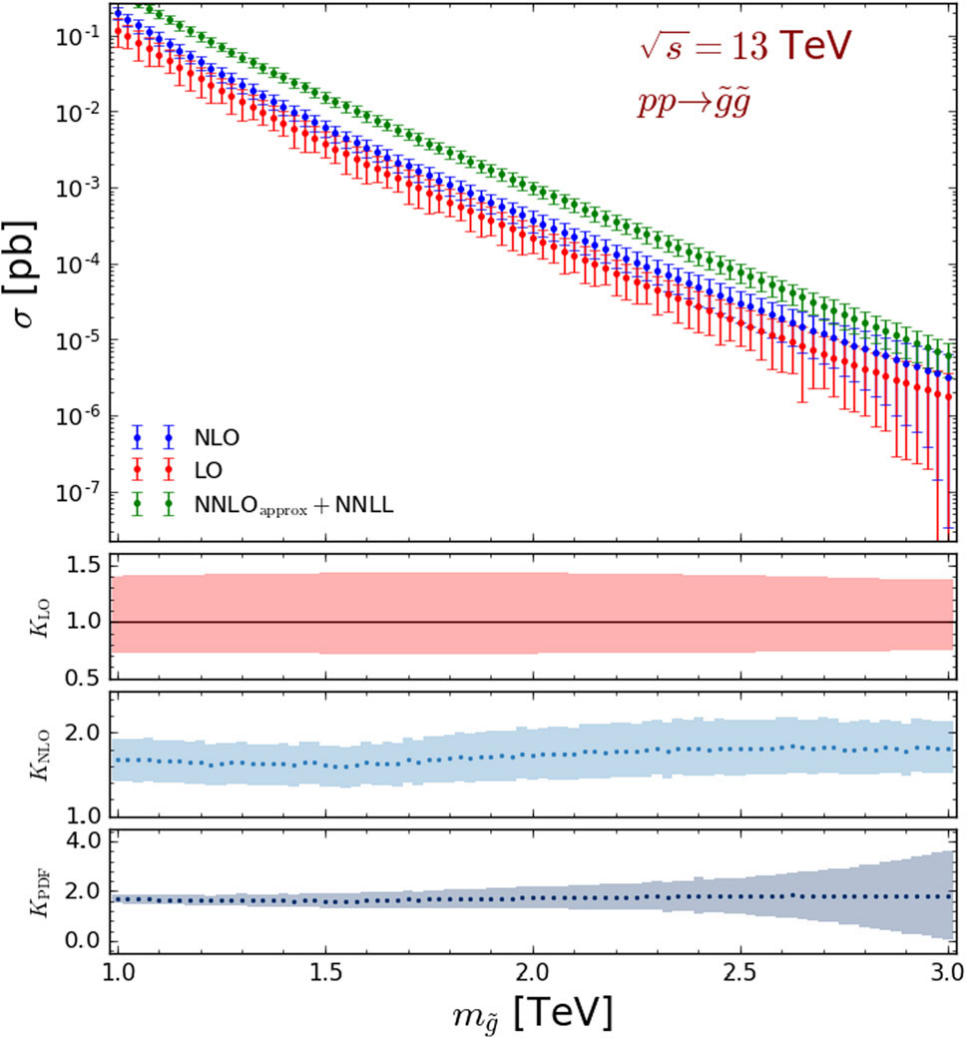
Total LO (red), NLO (blue) and NNLO$_\mathrm{approx}$+NNLL (green) cross sections (upper panel) and *K*-factors (three lower panels, where the results are normalised to the LO central value) for gluino pair-production, at a centre-of-mass energy of $\sqrt{s}=13$ TeV. In the upper panel, the error bands correspond to the quadratic sum of the scale and PDF uncertainties, whilst in the second and third panels, respectively, they refer to the scale uncertainties on the LO and NLO predictions. The last panel focuses on the PDF errors

In the upper panel of Fig. 2, we present the total LO (red) and NLO (blue) gluino pair-production cross section for gluino masses ranging from 1 to 3 TeV, the error bars being associated with the quadratic sum of the scale and PDF uncertainties. The cross section central value is found to vary within the 100–0.001 fb range when the gluino mass varies from 1 to 3 TeV, so that at least tens of gluino events could be expected even for a very heavy gluino benchmark at a high-luminosity upgrade of the LHC. We compare our predictions with the total rates traditionally employed by the ATLAS and CMS collaborations to extract gluino limits, as documented by the LHC Supersymmetry Cross Section Working Group [30]. Hence we include, in the first panel of Fig. 2, total gluino-pair production cross sections matching approximate fixed-order results at next-to-next-to-leading order and threshold-resummed predictions at the next-to-next-to-leading logarithmic accuracy (NNLO$_\mathrm{approx}$ + NNLL, in green). Following the PDF4LHC recommendations, those more accurate NNLO$_\mathrm{approx}$+NNLL predictions are obtained by convoluting the partonic cross section with a combination of NLO CTEQ6.6M [31] and MSTW2008 [32] densities. This choice, together with the impact of the higher-order corrections, leads to NNLO$_\mathrm{approx}$+NNLL results greater than our NLO predictions by a factor of about 2. While in the following we use NLO-accurate total rates (as the latter exist for any new physics model through a joint use of FeynRules [33], NLOCT [34] and MG5_aMC), we evaluate the impact of higher-order corrections whenever the relevant calculations exist, *i.e.* in this section and Sect. 4.

With the second and third panels of the figure, we emphasise the significant reduction of the scale uncertainties at NLO by depicting the LO and NLO scale uncertainty bands respectively, the $K_\mathrm{LO}$ and $K_\mathrm{NLO}$ quantities, presented in the two subfigures, these being the LO and NLO cross sections normalised to the LO central value. Such better control in the theoretical predictions is one of the main motivations for relying on NLO simulations instead of on LO ones. In the lower panel of Fig. 2, we focus on the PDF uncertainties associated with the total rates and present the $K_\mathrm{PDF}$ quantity where the NLO result (with its PDF error band) is again shown relatively to the LO central result. We omit the corresponding LO curve, as it is similar to the NLO one, the same PDF set being used both at LO and NLO in order to avoid having to deal with the poor-quality LO NNPDF 3.0 fit [23]. Whilst the uncertainties are under good control over most of the probed mass range, the poor PDF constraints in the large Bjorken-*x* regime lead to predictions plagued by sizeable uncertainties for gluino heavier than about 2.6–2.7 TeV. Finally, our results show that the NLO *K*-factor $K_\mathrm{NLO}$ is of about 1.6–1.7, a typical value for a strong supersymmetric production process, and features a significant gluino mass dependence. The latter originates from the quark–antiquark contributions to the cross section that become relatively larger with respect to the gluon fusion ones with increasing Bjorken-*x* values [35].

**Fig. 3:**
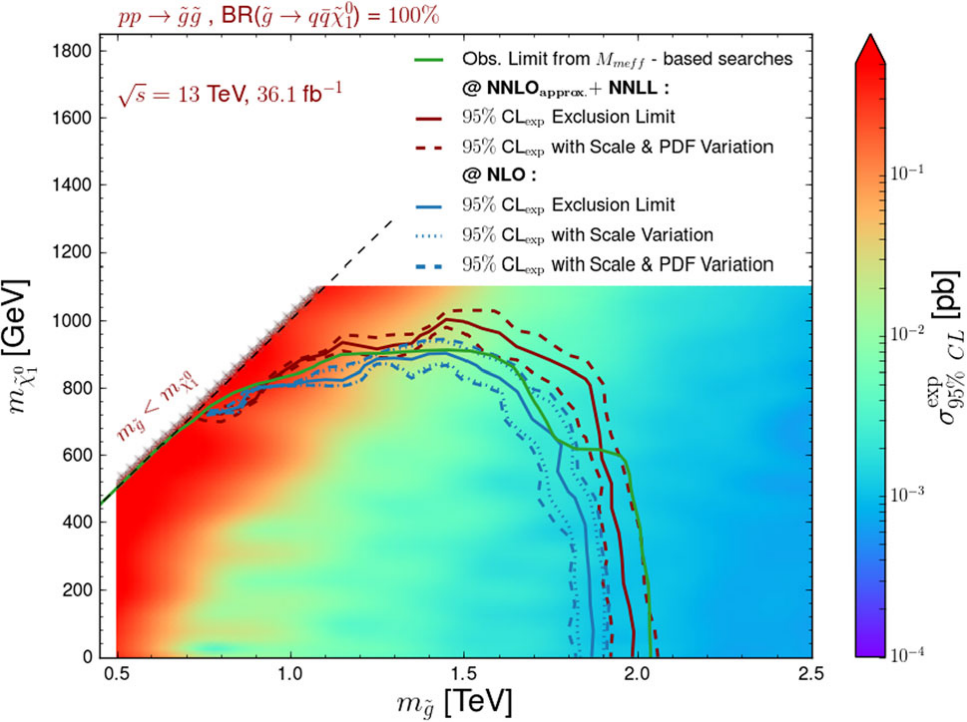
Constraints on the gluino-neutralino simplified model under consideration, represented as 95% confidence level exclusion contours in the $(m_{\tilde{g}}, m_{\tilde{\chi }_1^0})$ plane. We compare the exclusion obtained with the ATLAS-SUSY-2016-07 reimplementation in the MadAnalysis 5 framework [36] when normalising the signal to NLO (blue) and to NNLO$_\mathrm{approx}$+NNLL (red) with the official ATLAS results, extracted using the $M_\mathrm{eff}$ signal regions only [11] (solid green). Moreover, we include the uncertainty band on the MadAnalysis 5 results as originating from scale uncertainties (dotted) and from the quadratic combination of the scale and PDF uncertainties (dashed). The colour scheme represents the cross section value excluded at the 95% confidence level for each mass configuration

We then predict, for several $(m_{\tilde{g}},m_{\tilde{\chi }_1^0})$ configurations, how the signal events would populate the different signal regions of the ATLAS-SUSY-2016-07 search for supersymmetry [11]. In practice, we use the corresponding recast analysis as implemented in the MadAnalysis 5 public database [36], together with the appropriate Delphes 3 configuration for the simulation of the detector response. In this analysis, the ATLAS collaboration investigates the potential of a signature featuring multiple jets and missing transverse energy through two approaches. The first one relies on the so-called effective mass $M_\mathrm{eff}(N)$, a variable defined as the scalar sum of the transverse momenta of the *N* leading jets and the missing transverse energy. The second one is based on the recursive jigsaw reconstruction technique [37]. Whilst all $M_\mathrm{eff}$-based signal regions have been implemented in MadAnalysis 5, the recursive jigsaw reconstruction ones have been ignored due to the lack of information allowing for their proper recasting. They are thus ignored in the following study as well.

Our results are presented in Fig. 3 in the form of exclusion contours in the $(m_{\tilde{g}},m_{\tilde{\chi }_1^0})$ mass plane, to which we supplement the values of the signal cross section that are excluded at the 95% confidence level through a colour code. The exclusion contours and excluded cross sections at the 95% confidence level are extracted by means of Gaussian process regression with a conservative amount of data as implemented in the Excursion package [38].

We compare our predictions (the solid blue line), obtained with the setup described above, with the official ATLAS limits (the green line) as originating from the $M_\mathrm{eff}$-based signal region yielding the best expectation. ATLAS simulations are based on calculations at the LO accuracy in which samples of events describing final states featuring up to two extra jets are merged [39]. Moreover, the ATLAS results are normalised to NLO cross sections matched with threshold resummation at the next-to-leading logarithmic accuracy (NLO+NLL) [30]. The ATLAS setup therefore differs from ours both at the level of the differential distributions, as we model the properties of the second radiation jet solely at the level of the parton showers, and at the level of the total rates that are evaluated at the NLO matched with parton showers (NLO+PS) accuracy. This consequently results in MadAnalysis 5 limits slightly weaker than the ATLAS ones by about 10%, especially in the light neutralino mass regime.

**Fig. 4:**
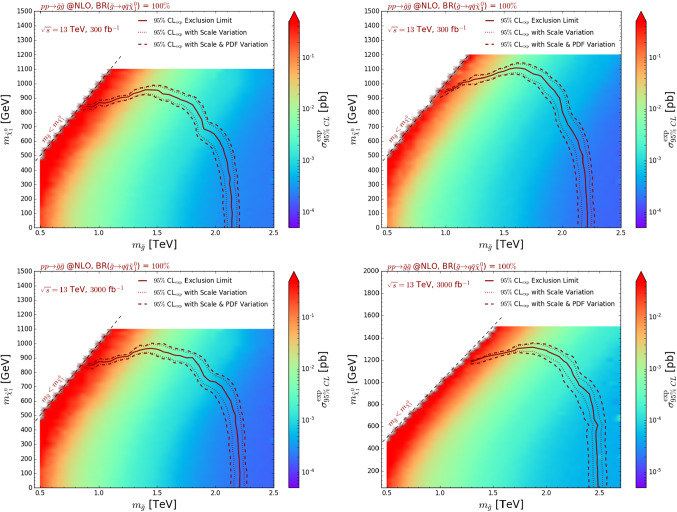
Expected constraints on the gluino-neutralino simplified model under consideration, represented as 95% confidence level exclusion contours in the $(m_{\tilde{g}}, m_{\tilde{\chi }_1^0})$ plane. We present the exclusions derived by extrapolating with MadAnalysis 5 the expectation of the ATLAS-SUSY-2016-07 analysis for 36 fb$^{-1}$ of LHC collisions to 300 fb$^{-1}$ (upper) and 3000 fb$^{-1}$ (lower). In the left panel, we extrapolate the uncertainties on the background linearly (*i.e.* the errors are assumed to be dominated by the systematics) while in the right panel, we extrapolate them proportionally to the square root of the luminosity (*i.e.* the errors are assumed to be dominated by statistics). The colour scheme represents the cross section value excluded at the 95% confidence level for each mass configuration

With the goal of assessing the importance of the signal normalisation, we extract bounds on the model by making use of NNLO$_\mathrm{approx}$+NNLL rates (red contour) instead of NLO ones (blue contour), NNLO$_\mathrm{approx}$+NNLL predictions being the most precise estimates for gluino-pair production to date. While still different from what has been used in the ATLAS study, NLO-NLL and NNLO$_\mathrm{approx}$+ NNLL predictions are known to be consistent with each other when theory error bands are accounted for. This has been documented, in the case of a gluino simplified model in which all squarks are decoupled, by the LHC Supersymmetry Cross Section Working Group.^4^ We observe a better agreement with the ATLAS results, showing the important role played by the new physics signal normalisation in the limit setting procedure. Large differences of about 5% on the mass limits are nevertheless still noticeable, showing that not only the normalisation but also the shape of the distributions are important ingredients. The ATLAS-SUSY-2016-07 analysis indeed relies on the $M_\mathrm{eff}(N)$ variable that is particularly sensitive to the modelling of the second jet, as $N\ge 2$ for all the analysis signal regions. In our setup in which NLO matrix elements are matched with parton shower, the second jet properties are described at the leading-logarithmic accuracy, the presence of this jet in the event final state solely originating from parton showering. This contrasts with ATLAS simulations in which LO matrix-element corrections are included as well, their final merged Monte Carlo signal samples including the contributions of LO matrix elements for gluino pair-production in association with two jets. This should motivate the usage of merged NLO samples matched with parton showers, so that predictions for observables sensitive to the sub-leading jet activity could be precisely achieved both for the shapes and the rates. The investigation of the actual impact of such an NLO multipartonic matrix element merging however goes beyond the scope of this work.

We also estimate in Fig. 3, the impact of the scale and PDF errors on the exclusion contours. For both MadAnalysis 5 predictions in which NLO (blue contour) and more precise NNLO$_\mathrm{approx}$+NNLL (red contour) are used for the signal normalisation, we describe the effect of the scale uncertainties through dotted contours and the one of the combined scale and parton density uncertainties through dashed contour. It turns out that the uncertainties on the signal impacts the gluino mass limits by about 50 GeV in both cases, the effect being mostly dominated by scale variations. The reach of the considered ATLAS-SUSY-2016-07 analysis concerns gluino masses smaller than about 1.8 TeV. This corresponds to a mass range where the uncertainty on the predictions is dominated by the scale variations, as shown in Fig. 2. The latter indeed shows that the PDF errors (lower panel of the figure) are at the level of a few percents for $m_{\tilde{g}} < 1.8$ TeV, the parton density fits being under a very good control for the corresponding Bjorken-*x* values.

In order to estimate the reach of this ATLAS supersymmetry search in the context of the future runs of the LHC, we make use of the framework detailed in Sect. 2.3 to extrapolate the results to 300 fb$^{-1}$ and 3000 fb$^{-1}$. As the ATLAS note of Ref. [11] does not include detailed and separate information on the systematical and statistical components of the uncertainties associated with the SM expectation in each signal region, we consider the two implemented options for their extrapolation to higher luminosities. More conservative, a linear extrapolation assumes that the error on the SM background is mostly dominated by its systematical component and scales proportionally to the luminosity (see the first term in Eq. (6)). More aggressive, an extrapolation in which the error scales proportionally to the square root of the luminosity (second term of Eq. (6)) considers that the background uncertainties are mainly of a statistical origin. The second option hence naively leads to a more important gain in sensitivity for higher luminosities, by definition. For all our predictions, we normalise the signal rates to NLO.

The results are presented in Fig. 4, first, by scaling the background uncertainties linearly to the luminosity (left panel, assuming that the background errors are dominated by the systematics), and second, by scaling them proportionally to the square root of the luminosity (right panel, assuming that the background errors are dominated by the statistical uncertainties). In all cases, we moreover assess the impact of the theory errors, the scale and PDF uncertainties being combined quadratically.

For an extrapolation to 300 fb$^{-1}$ (upper subfigures), the gluino mass limits are pushed to 2.1–2.2 TeV for a light bino-like neutralino with $m_{\tilde{\chi }_1^0} \lesssim 500$ GeV. The 36 fb$^{-1}$ exclusion is then found to be improved by about 15–20% (or 300–400 GeV). For such a mass range, the error on the theoretical predictions is still dominated by the scale variations (see Fig. 2) and only mildly impacts the exclusion, the effects reaching a level of about 5%. Such a small effect on a mass limit is related to the behaviour of the cross section with the increasing gluino mass, that is only reduced by a factor of a few. Comparing the left and right upper figures, one can assess the impact of the different treatment for the extrapolation of the background uncertainties. In the parameter space region under discussion, the impact is mild, reaching roughly a level of about 5% on the gluino mass limit. Such a small effect originates from the small resulting difference on the background error, that is 3 times smaller in the more aggressive case. Correspondingly, this allows us to gain a factor of a 3 in cross section, or equivalently a few hundreds of GeV in terms of a mass reach.

For more compressed scenarios in which the neutralino is heavier ($m_{\tilde{\chi }_1^0} > rsim 800$ GeV) and the gluino lighter ($m_{\tilde{g}} \in [1, 1.7]$ TeV), the treatment of the background extrapolation has a quite severe impact on the bounds on the neutralino mass. A more conservative linear extrapolation of the background error does not yield any significant change comparatively to the 36 fb$^{-1}$ case, neutralinos lighter than about 800 GeV being excluded. However, treating more aggressively the background uncertainties as being purely statistical, leads to an important increase in the bounds, neutralino masses ranging up to about 1 TeV becoming reachable. In those configurations, the spectra are more compressed and therefore more complicated to probe than for split configurations, consequently to the fact that the signal regions are less populated by the supersymmetry signals. A more precisely known background (with a relatively smaller uncertainty) is therefore crucial for being able to draw conclusive statements. As found in our results, any improvement, as little it is, can have a large impact.

In the lower subfigures, we present the results of an extrapolation to 3000 fb$^{-1}$. All above-described effects are emphasised to a larger extent. The differences in the treatment of the background uncertainties corresponding to knowing the background more accurately indeed now involve a factor of 10 in precision. A more interesting aspect concerns the theoretical predictions themselves that turn out to be known less and less precisely consequently to large parton density uncertainties. The limits indeed enter a regime in which large Bjorken-*x* are probed, which corresponds to PDF uncertainties contributing significantly to the total theory error. A better knowledge of the parton densities at large *x* and large scale is thus mandatory to keep our capacity to probe new physics in this regime.

**Fig. 5:**
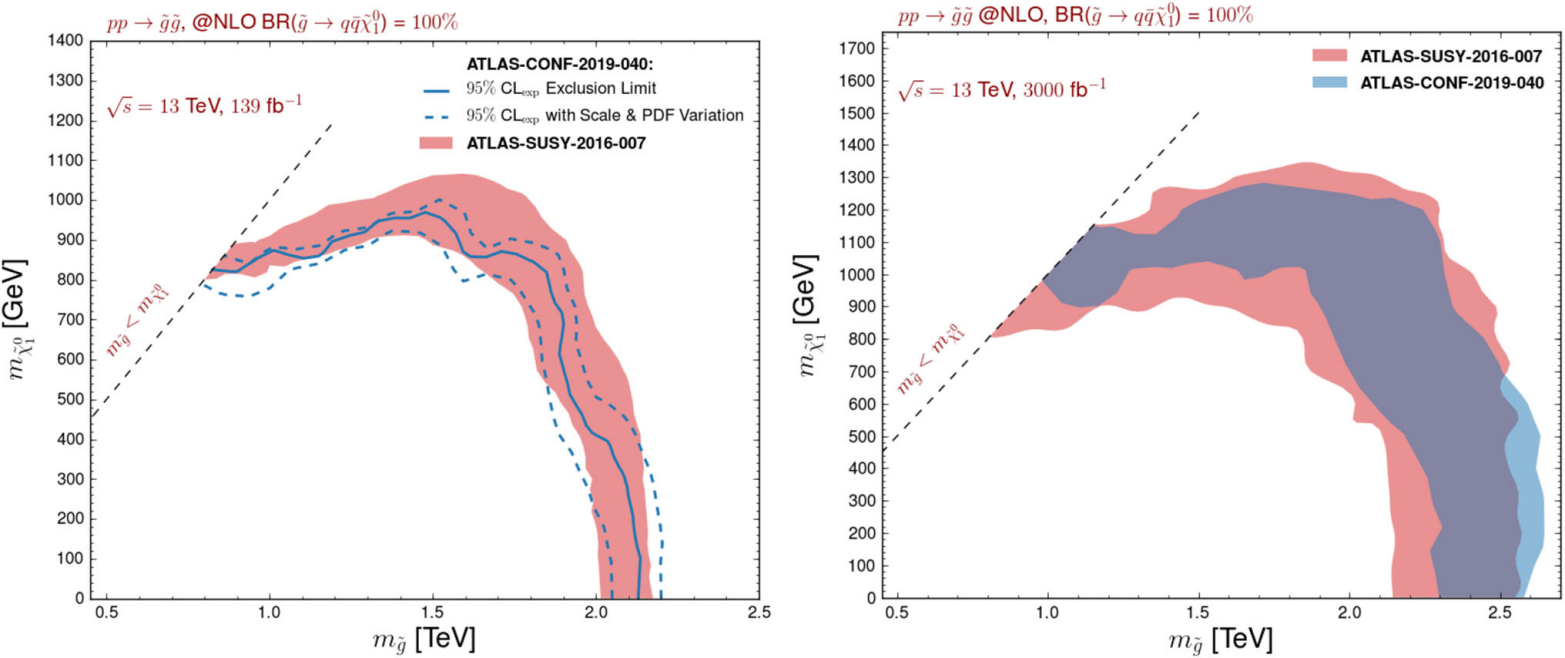
Expected constraints on the gluino-neutralino simplified model under consideration, represented as 95% confidence level exclusion contours in the $(m_{\tilde{g}}, m_{\tilde{\chi }_1^0})$ plane for 139 fb$^{-1}$ (left) and 3000 fb$^{-1}$ (right) of proton-proton collisions at a centre-of-mass energy of 13 TeV. We compare predictions obtained by recasting the results of the ATLAS-CONF-2019-140 analysis (blue lines), which we then extrapolate to 3000 fb$^{-1}$ (filled blue area), with those obtained by extrapolating the expectation of the ATLAS-SUSY-2016-07 analysis of 36 fb$^{-1}$ of LHC data to 139 fb$^{-1}$ and 3000 fb$^{-1}$ (solid red areas). The parameter space regions spanned by the various contours correspond to including both the PDF and scale uncertainties. The extrapolations are moreover performed conservatively (see the text)

We have verified that the obtained bounds were compatible with the naive extrapolations performed by the Collider Reach^5^ platform that extracts naive limits of a given collider setup with respect to the reach of a second collider setup, rescaling the results of the later by ratio of partonic luminosities. For instance, an 1.8 TeV gluino excluded with 36 fb$^{-1}$ of LHC collisions would correspond to a 2.4–2.7 TeV exclusion at 300 fb$^{-1}$. This is in fair agreement with our findings, after accounting for the fact that Collider Reach uses the NNPDF 2.3 set of parton densities [40], a set of parton distribution functions whose fit only includes 2010 and 2011 LHC data, so that important differences are expected, particularly for large *x*-values.

Whilst our extrapolations rely on the reinterpretation of an ATLAS analysis of 36 fb$^{-1}$ of LHC collisions, they are quite robust despite the small luminosity under consideration. Multijet plus missing transverse energy studies targeting a monojet-like topology (*i.e.* with a hard selection on the leading jet) are indeed limited by systematics [41], so that only mild improvements could be expected with a higher luminosity. This is what has been found in the results of Fig. 4, the bounds being improved by at most 20% in mass when going from 300 to 3000 fb$^{-1}$. This subsequently also implies that the expected sensitivity should be rather independent of the initially-analysed luminosity. We further demonstrate those considerations in Fig. 5.

In the left panel of the figure, we extrapolate the results of the ATLAS-SUSY-2016-07 analysis to the full Run 2 luminosity of 139 fb$^{-1}$, the theory errors being combined quadratically. In our extrapolation procedure, we have considered both that the background uncertainties are dominated by the systematics (linear scaling) and by the statistics (scaling proportional to the square root of the luminosity). The two set of results have been merged and presented as the unique envelope of the exclusion bands derived from the two extrapolation procedures. They could hence be seen as a conservative theory estimate for the LHC sensitivity at 139 fb$^{-1}$, when estimated from official 36 fb$^{-1}$ results.

The ATLAS-SUSY-2016-07 analysis has been updated last summer as the ATLAS-CONF-2019-040 analysis [12], so that the most recent and stringent ATLAS limits on the considered gluino simplified model now encompass the analysis of the full LHC Run 2 dataset. On the other hand, the updated analysis has been recently added to the PAD [42], so that it can be used within the MadAnalysis 5 framework for reinterpretation studies. The corresponding 95% confidence level contour is shown on the left panel of Fig. 5 (solid blue line), together with the uncertainty band stemming from combining the scale and PDF uncertainties in quadrature. In addition, we also present predictions for the bounds as obtained from an extrapolations of early Run 2 results focusing on 36 fb$^{-1}$ of LHC data. After accounting for the error bands, the two sets of constraints are found in good agreement, as expected.

On the right panel of Fig. 5, we consider the two ATLAS multijet plus missing transverse energy analyses that have been above-mentioned, namely the early LHC Run 2 ATLAS-SUSY-2016-07 analysis (36 fb$^{-1}$, red) and the full Run 2 ATLAS-CONF-2019-040 analysis (139 fb$^{-1}$, blue). We reinterpret their results with MadAnalysis 5, and extrapolate the predictions that have been obtained for the nominal luminosities of the two analyses to 3000 fb$^{-1}$. The contours shown on the figure are obtained as before, *i.e.* by considering independent scalings of the background assuming that it is either dominated by the systematical or by the statistical uncertainties. The envelopes of the two exclusion bands (including the theory errors) are then reported in the figure. The two solid areas presented on the figure are found to largely overlap and be consistent with each other.

**Fig. 6:**
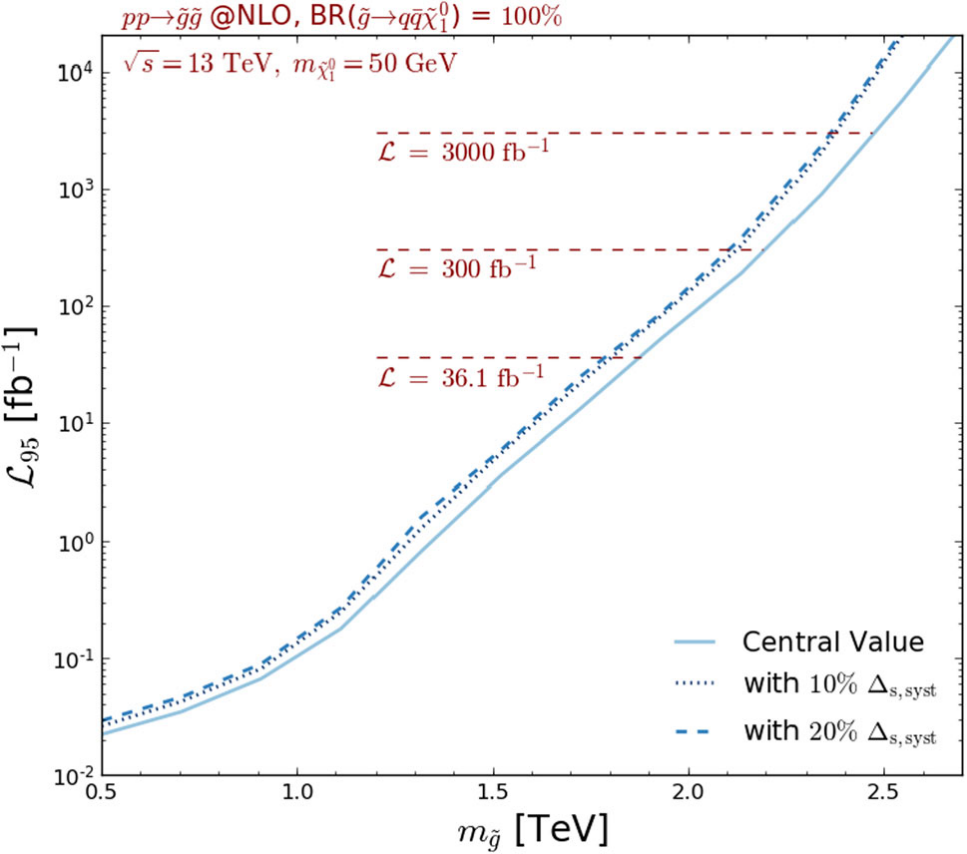
Luminosity necessary to exclude, at the 95% confidence level, a given gluino-neutralino new physics setup with the ATLAS-SUSY-16-07 analysis. We fix the neutralino mass to $m_{\tilde{\chi }_1^0}=50$ GeV, assume that the uncertainties on the background are dominated by their statistical component, and include systematical uncertainties on the signal of 0% (solid line), 10% (dotted line) and 20% (dashed line)

In Fig. 6, we make use of the MadAnalysis 5 infrastructure to estimate, for various benchmark points, the luminosity $\mathcal{L}_{95}$ that is required to exclude the scenario at the 95% confidence level. We still consider the ATLAS-SUS-2016-07 analysis, fix the neutralino mass to 50 GeV and let the gluino mass vary. We compute $\mathcal{L}_{95}$ for two choices of systematics on the signal (combined in both cases with the theory errors quadratically), namely 10% (dotted line) and 20% (dashed line), and compare the predictions with the central value where the signal is perfectly known (solid line). In those calculations, we scale the error on the background proportionally to the square root of the luminosity, as if it was mainly dominated by its statistical component. Our analysis first shows that light gluinos with masses smaller than about 1.5 TeV can be excluded with a luminosity $\mathcal{L}_{95}$ of a few fb$^{-1}$, as confirmed by the early Run 2 ATLAS search of Ref. [43] that consists of the 3.2 fb$^{-1}$ version of the ATLAS-SUSY-2016-07 analysis. The steep fall of the cross section with an increasing gluino mass moreover implies that the high-luminosity LHC reach of the analysis under consideration will be limited to gluinos of about 2.5 TeV, a bound that could be reduced by about 10% if the systematics on the signal are of about 10–20%. This order of magnitude has been found to agree with older ATLAS estimates [12].

**Fig. 7:**
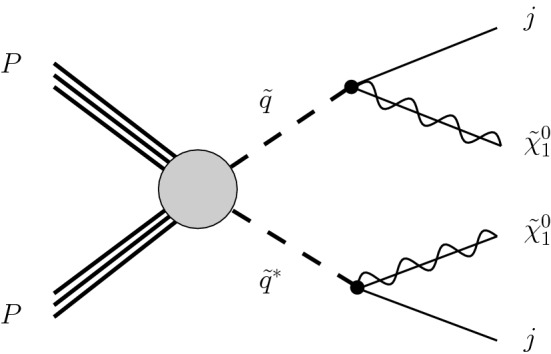
Generic Feynman diagram associated with the production and decay of a pair of squarks in the considered MSSM-inspired squark simplified model. The figure has been produced with the help of the JaxoDraw package [21]

## Squark and neutralino mass limits

In this section, we consider a second class of simplified models inspired by the MSSM that is widely studied in the context of the LHC searches for new physics. As in Sect. 3, all superpartners, except for two under investigation, are decoupled. This time, these are taken to be a squark and the lightest neutralino. In practice, we hence supplement the SM field content by one species of first generation squark $\tilde{q}$ and the lightest neutralino $\tilde{\chi }_1^0$, assumed to be bino-like. In this configuration, squarks can be pair-produced through standard QCD interactions, and then each decays into the lightest neutralino and an up quark, as illustrated by the generic Feynman diagram of Fig. 7. Such a parton-level final state comprised of two quarks and two invisible neutralinos therefore manifests itself, after parton showering and hadronisation, as a multijet plus missing transverse energy topology.

The ATLAS analyses considered in Sect. 3, targeting multijet plus missing energy signs of new physics, are therefore appropriate to put constraints on the model under consideration. Those analyses indeed include not only signal regions dedicated to probe final state featuring a large jet multiplicity (that are thus ideal to target the previously considered gluino simplified model), but also include signal regions targeting signals exhibiting a smaller jet multiplicity (that are thus excellent probes for the present squark simplified model). In the following, we only make use of the most recent search, ATLAS-CONF-2019-140 [12].

**Fig. 8:**
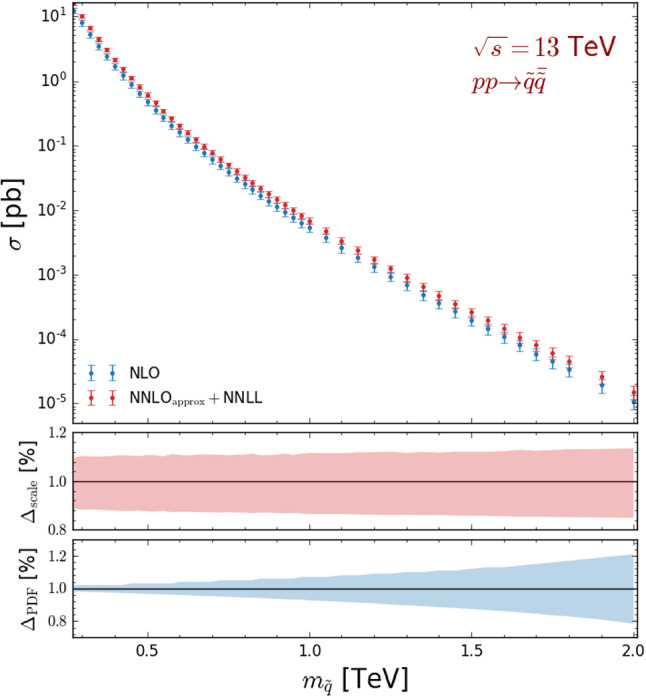
Total NLO (blue) and approximate NNLO+NNLL (red) cross section (upper panel) for squark pair production in proton-proton collisions at a centre-of-mass energy of 13 TeV. The error bars represent the quadratic sum of the scale and PDF uncertainties. In the middle and lower panels of the figure, we report the NLO scale and PDF uncertainties respectively, after normalising the results to the central NLO cross section value

**Fig. 9:**
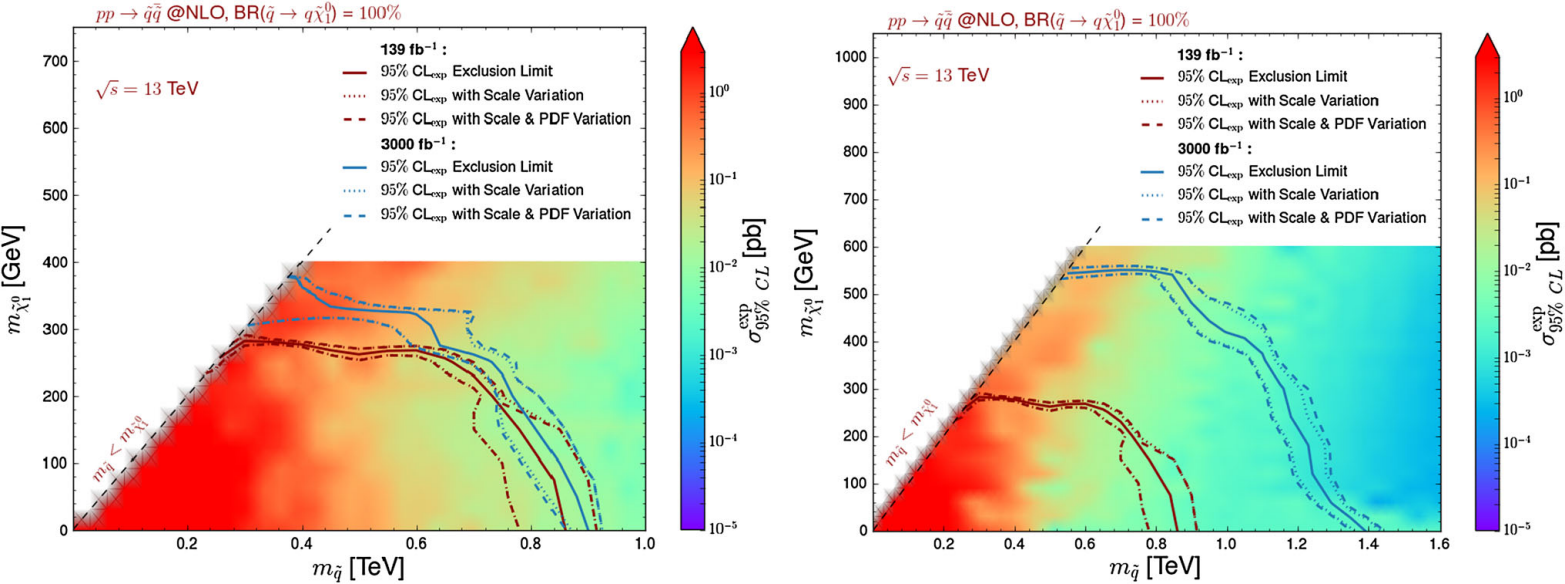
Expected constraints on the squark-neutralino simplified model under consideration, represented as 95% confidence level exclusion contours in the $(m_{\tilde{q}}, m_{\tilde{\chi }_1^0})$ plane for 139 fb$^{-1}$ (red) and 3000 fb$^{-1}$ (blue) of proton–proton collisions at a centre-of-mass energy of 13 TeV. We derive those bounds with the ATLAS-CONF-2019-140 implementation in MadAnalysis 5 and extrapolate the uncertainties on the background as if they are systematically-dominated (left, scaling proportional to the luminosity ) or statistically-dominated (right, scaling proportional to the square root of the luminosity)

Making use of the same simulation setup as in Sect. 3, we study the LHC sensitivity to this model after the full Run 2 and present the expectation of its high-luminosity operation run. Our results are derived from simulations at the NLO+PS accuracy, so that our signal samples are normalised at the NLO accuracy. The rates that we employ in the following are depicted in the upper panel of Fig. 8, where we show total NLO-accurate squark-pair production cross sections as returned by MG5_aMC when using the MSSM implementation developed in Ref. [7] and the NLO set of NNPDF 3.0 parton densities [23] (blue). Predictions are given for squark masses ranging from 250 GeV to 2 TeV and include theory errors that we estimate by adding scale and PDF uncertainties in quadrature. Those uncertainties are further described more precisely in the middle and lower panels of the figure, where they are given after normalising the results to the central NLO cross section value for each mass point.

We obtain cross sections that vary from 10 pb for $m_{\tilde{q}}\sim 250$ GeV to 0.01 fb for 2 TeV squarks. They are two orders of magnitude lower than in the gluino case for a specific mass value, as expected from the fact that squarks are scalars and are colour triplets and not octets. Scale uncertainties are found to be independent of the squark mass for the considered $m_{\tilde{q}}$ range, and are of about 15% (middle panel of the figure). In contrast, the PDF errors strongly depend on the squark mass $m_{\tilde{q}}$ (lower panel of the figure), as they are correlated with the associated Bjorken-*x* regime. They are of a few percents and thus subleading for small $m_{\tilde{q}}$ values, and grow for increasing squark masses, eventually reaching 20% for $m_{\tilde{q}} = 2$ TeV. For larger and larger *x*-values (and thus larger and larger $m_{\tilde{q}}$), the quark-antiquark contributions to the cross section play a bigger and bigger role. Simultaneously, the impact of the Bjorken-*x* regime in which the PDF sets are more poorly constrained by data gets more important.

As in Sect. 3, we compare our predictions to the cross section values usually employed by the LHC collaborations (red curve), as reported by the LHC Supersymmetry Cross Section Working Group [30]. The latter are however only provided for a simplified model in which all squarks except the two stop squarks are mass-degenerate. We therefore normalise the NNLO$_\mathrm{approx}$+NNLL results by an extra factor of 1/10, which should be a fair enough approximation for small squark masses. Nevertheless, as both NLO and NNLO$_\mathrm{approx}$+NNLL predictions are consistent, we consider (exact) NLO rates in the following.

In Fig. 9, we reinterpret the results of the ATLAS-CONF-2019-040 analysis with MadAnalysis 5 and present the expected exclusion contours both at the nominal luminosity of 139 fb$^{-1}$, after extrapolating the findings to 3000 fb$^{-1}$, using for each point the region yielding the best expected sensitivity. Neutralino masses below about 300 GeV are currently (*i.e.* for a luminosity of 139 fb$^{-1}$) excluded, for squark masses ranging up to about 900 GeV. This may seem to contrast by a factor of about 2 with the current bounds on this class of simplified model set by the ATLAS collaboration [12]. This is however not surprising as the collaboration only interprets its results for a simplified model in which the superpartner spectrum exhibits 10 mass-degenerate left-handed and right-handed squarks (*i.e.* all squarks except the two stop squarks are degenerate). The corresponding signal cross sections are therefore about 10 times larger, so that much stronger limits could be extracted. In comparison with final Run 2 CMS results [44, 45] for which result interpretations both for eight mass-degenerate squarks and a single squark species are provided, we obtain more conservative bounds that are roughly 20% weaker in terms of excluded masses. When accounting for the uncertainty bands, our predictions agree with the experimental findings, as the uncertainty bands overlap.

Extrapolating the results to a luminosity of 3000 fb$^{-1}$, *i.e.* expected luminosity of the high-luminosity phase of the LHC, we obtain expected bounds which are improved quite a bit. The magnitude of the improvement is found strongly related to how the background uncertainties will be controlled, as visible by comparing the curves corresponding to 3000 fb$^{-1}$ (blue) in the two panels of the figure. Assuming that the background is dominated by the systematics or the statistics change the results by more than 40%.

## Sensitivity to simplified *s*-channel dark matter models

In this section, we investigate the sensitivity of the LHC to a simplified dark matter (DM) model. We assume that DM is described by a massive Dirac fermionic particle *X* that communicates with the Standard Model through the exchange of a spin-1 mediator *Y*. Motivated by models with an extended gauge group, we consider that the mediator couples only either to a pair of DM particles, or to a pair of SM fermions. Such a configuration is typical from the so-called *s*-channel dark matter models [13, 14]. In this class of scenarios, DM can only be pair-produced at colliders, from the scattering of a pair of SM quarks and through the *s*-channel exchange of the mediator.

The corresponding Lagrangian can generically be written as

8 $$\begin{aligned} \mathcal{L}= & {} \ \mathcal{L}_\mathrm{SM} + \mathcal{L}_\mathrm{kin} + \bar{X} \gamma _\mu \big [g^V_X + g^A_X \gamma _5 \big ] X\ Y^\mu \nonumber \\&\ \ + \sum _q \Big \{\bar{q} \gamma _\mu \big [ g^V_q + g^A_q \gamma _5 \big ] q \Big \} Y^\mu \end{aligned}$$

where $\mathcal{L}_\mathrm{SM}$ refers to the SM Lagrangian and $\mathcal{L}_\mathrm{kin}$ contains gauge-invariant kinetic and mass terms for all new fields. The next term includes the vector and axial-vector interactions of the mediator with DM, their strength being denoted by $g^V_X$ and $g^A_X$ respectively, and the last term focus on the mediator interactions with the SM quarks. The latter are assumed universal and flavour-independent, their strength being $g^V_q$ and $g^A_q$ in the vector and axial-vector case respectively, regardless of the quark flavour.

In our analysis, we focus on two further simplified scenarios originating from that model. In a first case (that we label **S1**), the mediator couplings are taken as of a vectorial nature, whilst in the second case (that we label **S2**), they are taken as of an axial-vectorial nature. In other words, the two scenarios are defined as

9 $$\begin{aligned} \mathbf{S1}:\ g_q^A = g_X^A = 0\ ; \qquad \mathbf{S2}:\ g_q^V = g_X^V = 0. \end{aligned}$$

In order to study the sensitivity of the LHC to these two classes of scenarios, we make use of the publicly available^6^ implementation of the model in the FeynRules package [33] introduced in Ref. [13], as well as of the corresponding public UFO [46] library. As in the previous sections, hard scattering events are generated at the NLO accuracy in QCD with MG5_aMC [22] and then matched with parton showering and hadronisation as performed by Pythia [27]. In our simulations, the matrix elements are convoluted with the NLO set of NNPDF 3.0 parton densities [23]. We derive the LHC sensitivity to the model by considering the associated production of a pair of dark matter particles with jets, a signature targeted by the ATLAS-CONF-2019-040 analysis [12] introduced in the previous sections. This ATLAS study searches for new phenomena in a luminosity of 139 fb$^{-1}$ of LHC data at a centre-of-mass energy of 13 TeV, investigating events featuring at least two hard jets and a potential subleading jet activity.

**Fig. 10:**
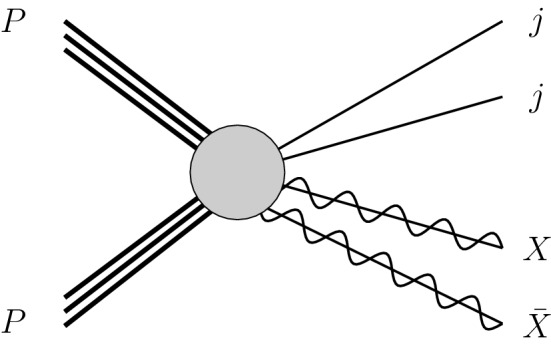
Generic Feynman diagram associated with the production of a pair of dark matter particles *X* in association with two hard jets. The figure has been produced with the help of the JaxoDraw package [21]

As the analysis selection requires at least two very hard jets, we consider as a hard-scattering process the production of a pair of DM particles with two hard jets, as sketched in Fig. 10. Moreover, we impose two conservative (with respect to the ATLAS analysis) generator-level selections. We constrain the transverse momentum of the hardest of the jets to satisfy $p_T>150$ GeV, and the parton-level missing transverse energy (*i.e.* the transverse energy of the vector sum of the transverse momenta of the two DM particles) to fulfil  GeV. Moreover, the reference renormalisation and factorisation scales are set to the mass of the mediator $m_Y$, and we estimate the associated uncertainties as usual, by independently varying the two scales by a factor of 2 up and down around the central scale choice.

We begin with considering a series of scenarios featuring light dark matter, *i.e.* with a dark matter mass $m_X$ fixed to 100 GeV. The mediator mass is kept free to vary in the [0.3, 2] TeV range. In Table 1, we present the sensitivity of the ATLAS-CONF-2019-040 analysis to those scenarios, both at the nominal luminosity of 139 fb$^{-1}$ and for the high-luminosity LHC run (with 3000 fb$^{-1}$). For each spectrum configuration, we show NLO signal cross sections (second and fifth columns for the **S1** and **S2** benchmarks respectively), as obtained following the simulation setup described above and for couplings obeying to Eq. (9). Moreover, those predictions are obtained after fixing the remaining non-vanishing free parameters to the reference values

10 $$\begin{aligned} \mathbf{S1}:&\ g_q^V = 0.25\ ,\ g_X^V = 1; \nonumber \\ \mathbf{S2}:&\ g_q^A = 0.25\ ,\ g_X^A = 1, \end{aligned}$$

which consist in one of the benchmarks studied by the LHC dark matter working group [14].

**Table 1:** Expected constraints on various light dark matter *s*-channel scenarios. The dark matter mass is fixed to $m_X=100$ GeV and the couplings satisfy Eq. (9). Reference NLO cross sections (second and fifth columns) are provided for a case where the remaining free couplings are set as in Eq. (10), and can be compared with the 95% confidence level limits expected from the reinterpretation of the ATLAS-CONF-2019-040 analysis of (third and sixth columns). Our results are present both at the nominal luminosity of 139 fb$^{-1}$ and after being extrapolated to 3000 fb$^{-1}$ assuming systematics-dominated uncertainties (in parentheses). Those bounds are also translated into a bound on the couplings for a $g_q=g_X$ configuration (fourth and seventh columns)

$m_{Y}$ [TeV]	Vector couplings (**S1**)			Axial-vector couplings (**S2**)		
	$$\sigma _\mathrm{NLO}$$ [pb]	$$\sigma _{95} $$ [pb]	$$|g_{95}| $$	$$\sigma _\mathrm{NLO}$$ [pb]	$$\sigma _{95}$$ [pb]	$$|g_{95}|$$
0.3	$19.45^{+35.4\%\ +1.2\%}_{-77.1\%\ -1.2\%}$	1.553(1.318)	$0.532^{+0.06}_{-0.07} \left( 0.510^{+0.06}_{-0.07}\right)$	$9.21^{+30.9\%\ +1.2\%}_{-68.4\%\ -1.2\%}$	1.015(0.700)	$0.576^{+0.06}_{-0.07} \left( 0.525^{+0.05}_{-0.06}\right)$
0.5	$12.19^{+15.5\%\ +1.1\%}_{-39.3\%\ -1.1\%}$	0.667(0.568)	$0.484^{+0.02}_{-0.04} \left( 0.465^{+0.02}_{-0.04}\right)$	$9.73^{+15.8\%\ +1.3\%}_{-39.8\%\ -1.3\%}$	0.643(0.545)	$0.507^{+0.02}_{-0.04} \left( 0.486^{+0.02}_{-0.04}\right)$
0.7	$7.05^{+10.3\%\ +1.2\%}_{-29.5\%\ -1.2\%}$	0.368(0.311)	$0.478^{+0.01}_{-0.03} \left( 0.458^{+0.01}_{-0.03}\right)$	$6.41^{+9.2\%\ +1.3\%}_{-27.3\%\ -1.3\%}$	0.333(0.285)	$0.477^{+0.01}_{-0.03} \left( 0.459^{+0.01}_{-0.03}\right)$
0.8	$5.37^{+8.3\%\ +1.4\%}_{-25.8\%\ -1.4\%}$	0.312(0.266)	$0.491^{+0.01}_{-0.03} \left( 0.472^{+0.01}_{-0.03}\right)$	$5.22^{+6.3\%\ +1.2\%}_{-22.0\%\ -1.2\%}$	0.278(0.234)	$0.480^{+0.01}_{-0.02} \left( 0.460^{+0.01}_{-0.02}\right)$
0.9	$4.15^{+6.1\%\ +1.3\%}_{-21.7\%\ -1.3\%}$	0.242(0.169)	$0.491^{+0.01}_{-0.02} \left( 0.449^{+0.01}_{-0.02}\right)$	$4.13^{+5.5\%\ +1.3\%}_{-18.9\%\ -1.3\%}$	0.241(0.205)	$0.491^{+0.01}_{-0.02} \left( 0.472^{+0.01}_{-0.02}\right)$
1.0	$3.30^{+5.2\%\ +1.6\%}_{-20.0\%\ -1.6\%}$	0.224(0.189)	$0.511^{+0.01}_{-0.02} \left( 0.490^{+0.01}_{-0.02}\right)$	$3.39^{+4.7\%\ +1.6\%}_{-17.0\%\ -1.6\%}$	0.221(0.188)	$0.505^{+0.01}_{-0.02} \left( 0.485^{+0.01}_{-0.02}\right)$
1.2	$2.16^{+4.0\%\ +1.7\%}_{-16.6\%\ -1.7\%}$	0.204(0.174)	$0.554^{+0.01}_{-0.02} \left( 0.533^{+0.01}_{-0.02}\right)$	$2.17^{+3.9\%\ +1.7\%}_{-15.0\%\ -1.7\%}$	0.175(0.148)	$0.533^{+0.01}_{-0.02} \left( 0.511^{+0.01}_{-0.02}\right)$
1.4	$1.44^{+3.7\%\ +2.3\%}_{-13.5\%\ -2.3\%}$	0.139(0.118)	$0.557^{+0.01}_{-0.02} \left( 0.535^{+0.01}_{-0.02}\right)$	$1.42^{+2.5\%\ +1.9\%}_{-11.1\%\ -1.9\%}$	0.142(0.120)	$0.562^{+0.00}_{-0.01} \left( 0.539^{+0.00}_{-0.01}\right)$
1.5	$1.15^{+2.9\%\ +2.1\%}_{-11.9\%\ -2.1\%}$	0.139(0.117)	$0.590^{+0.01}_{-0.02} \left( 0.566^{+0.01}_{-0.02}\right)$	$1.15^{+2.6\%\ +2.3\%}_{-11.0\%\ -2.3\%}$	0.127(0.108)	$0.576^{+0.01}_{-0.02} \left( 0.554^{+0.00}_{-0.01}\right)$
1.8	$0.63^{+2.1\%\ +2.5\%}_{-8.5\%\ -2.5\%}$	0.121(0.103)	$0.662^{+0.01}_{-0.01} \left( 0.636^{+0.01}_{-0.01}\right)$	$0.66^{+1.9\%\ +2.6\%}_{-7.8\%\ -2.6\%}$	0.133(0.112)	$0.672^{+0.01}_{-0.01} \left( 0.643^{+0.01}_{-0.01}\right)$
2.0	$0.44^{+2.1\%\ +2.9\%}_{-8.6\%\ -2.9\%}$	0.104(0.089)	$0.699^{+0.01}_{-0.02} \left( 0.671^{+0.01}_{-0.01}\right)$	$0.44^{+1.6\%\ +3.1\%}_{-6.4\%\ -3.1\%}$	0.095(0.081)	$0.680^{+0.01}_{-0.01} \left( 0.653^{+0.01}_{-0.01}\right)$

We first assess the LHC sensitivity to each point for the two considered luminosities in terms of the signal cross section that is reachable at the LHC $\sigma _{95}$ (third and sixth columns of table 1 for the **S1** and **S2** benchmarks respectively) by reinterpreting, with MadAnalysis 5, the results of the ATLAS-CONF-2019-040 analysis. Second, we translate the cross section limits that we have obtained into a bound on a universal new physics coupling strength $g_{95}$ that is defined for scenarios in which

11 $$\begin{aligned} g_q=g_X\ . \end{aligned}$$

Moreover, we provide the $g_{95}$ limits together with the theory uncertainty stemming from scale and PDF variations (fourth and seventh column of the table). The most stringent bounds on the model originate from a single signal region of the analysis in which, the effective mass $M_\mathrm{eff}$ is imposed to be larger than 2.2 TeV. Such a cut is applied together with looser cuts on the jet properties, as compared with other signal regions featuring smaller effective masses.

For fixed vector couplings (**S1** scenarios), the NLO cross section $\sigma _\mathrm{NLO}$ decreases when the mediator mass increases and spans a range extending from about 450 fb for heavy mediators with a mass of about 2 TeV, to more than 10 pb for mediators lighter than 500 GeV. Those values and this steeply-falling behaviour are mainly driven by the heavy mass of the mediator as compared with the small dark matter mass. Larger cross sections are indeed obtained for smaller mediator masses as we lie closer to the resonant regime in which $m_Y \sim 2 m_X$. The cross section that is expected to be excluded at the 95% confidence level also falls down with $m_Y$, although the slope is much flatter. Moreover, $\sigma _{95} < \sigma _\mathrm{NLO}$. Consequently, all scenarios defined by the coupling assumptions of Eqs. (9) and (10) are excluded, already with the present full Run 2 luminosity.

Relaxing the coupling definitions of Eq. (10) and replacing it by the universal coupling constraint of Eq. (11), it turns out that couplings of 0.4–0.7 are excluded over the entire mass range, the best limits being obtained for scenarios featuring sub-TeV mediators and a spectrum such that one lies far enough from the resonant regime. In the latter case, the analysis is less sensitive as a consequence of the associated softer final state objects populating the signal events. The overall weak dependence of the excluded coupling on the mediator mass stems from various interplaying effects. First, the cross section has a quartic dependence on the couplings, so that a small coupling change leads to a large modification of the cross section. Second, there is a strong interplay between the mediator mass and the dark matter mass (*i.e.* if ones lies for enough from the resonant regime) and the kinematical configuration probed by the analysis cuts, especially for light mediators.

**Table 2:** Same as Table 1, but for a scenario in which $m_X$ is free and $m_Y$ has been set to 1.5 TeV

$m_{X}$ [GeV]	Vector coupling (**S1**)			Axial-vector coupling (**S2**)		
	$\sigma _\mathrm{NLO}$ [pb]	$\sigma _{95}$ [pb]	$|g_{95}|$	$\sigma _\mathrm{NLO}$ [pb]	$\sigma _{95}$ [pb]	$|g_{95}|$
200	$1.19^{+3.0\%\ +2.1\%}_{-12.2\%\ -2.1\%}$	0.149(0.126)	$0.595^{+0.006}_{-0.02} \left( 0.571^{+0.005}_{-0.02}\right)$	$1.11^{+2.5\%\ +2.2\%}_{-10.7\%\ -2.2\%}$	0.148(0.125)	$0.605^{+0.005}_{-0.02} \left( 0.580^{+0.005}_{-0.01}\right)$
350	$1.17^{+3.0\%\ +2.2\%}_{-12.5\%\ -2.2\%}$	0.115(0.098)	$0.560^{+0.005}_{-0.02} \left( 0.538^{+0.005}_{-0.02}\right)$	$0.85^{+2.5\%\ +2.1\%}_{-10.1\%\ -2.1\%}$	0.129(0.109)	$0.624^{+0.005}_{-0.02} \left( 0.598^{+0.005}_{-0.01}\right)$
500	$1.10^{+3.5\%\ +2.2\%}_{-12.6\%\ -2.2\%}$	0.143(0.122)	$0.601^{+0.006}_{-0.02} \left( 0.577^{+0.006}_{-0.02}\right)$	$0.51^{+2.7\%\ +2.2\%}_{-10.6\%\ -2.2\%}$	0.135(0.114)	$0.715^{+0.006}_{-0.02} \left( 0.685^{+0.006}_{-0.02}\right)$
650	$0.82^{+3.2\%\ +2.1\%}_{-13.2\%\ -2.1\%}$	0.149(0.127)	$0.653^{+0.006}_{-0.02} \left( 0.627^{+0.006}_{-0.02}\right)$	$0.15^{+2.7\%\ +2.4\%}_{-9.7\%\ -2.4\%}$	0.143(0.121)	$0.982^{+0.009}_{-0.02} \left( 0.941^{+0.009}_{-0.02}\right)$
800	$0.006^{+3.3\%\ +2.8\%}_{-13.8\%\ -2.8\%}$	0.131(0.110)	$2.171^{+0.02}_{-0.07} \left( 2.075^{+0.02}_{-0.07}\right)$	$0.0009^{+2.3\%\ +3.2\%}_{-11.4\%\ -3.2\%}$	0.121(0.104)	$3.456^{+0.03}_{-0.10} \left( 3.322^{+0.03}_{-0.09}\right)$
900	$0.001^{+3.5\%\ +3.5\%}_{-14.7\%\ -3.5\%}$	0.107(0.091)	$2.986^{+0.04}_{-0.10} \left( 2.863^{+0.04}_{-0.10}\right)$	$0.0002^{+3.1\%\ +3.5\%}_{-13.4\%\ -3.5\%}$	0.110(0.093)	$4.600^{+0.06}_{-0.15} \left( 4.412^{+0.05}_{-0.14}\right)$

In the heavy-mediator regime, considering **S2** scenarios featuring axial-vector mediator couplings leads to very similar results. In this limit, the relevant matrix elements are insensitive to the mediator nature. On the contrary, when one approaches the resonant regime, significant changes arise: The cross section turns out to be suppressed relatively to the vector **S1** scenario. This originates from the impact of the threshold regime that plays a larger and larger role for smaller and smaller masses. At threshold, the pair of dark matter particles is organised into a $^3P_1$ state, and not into a $^3S_1$ configuration as in the **S1** scenario. Consequently, signal cross sections are relatively suppressed. The small increase in cross section for low $m_Y$ values in the **S2** case hence stems from those threshold effects that are more and more tamed when one gets further from threshold, as well as from the cut on the leading jet of 150 GeV. As in the **S1** scenario, the entire mass range is excluded by the ATLAS-CONF-2019-040 analysis, which translates in the exclusion of couplings in the 0.4–0.7 ballpark for the considered mediator mass range.

Finally, those bounds are expected to only be sightly improved, by about 4–9%, after including 3000 fb$^{-1}$ of data for both scenarios. This is related to the systematical dominance of the uncertainties on the background, as we have chosen to scale it under that assumption, so that more luminosity will not bring much compared with the Run 2 results. Moreover, we observe that the results are plagued by quite modest theoretical uncertainties at the $g_{95}$ level (by virtue of the quartic dependence of the matrix element of the coupling).

In Table 2, we consider a new class of scenarios. This time, the mediator mass $m_Y$ is fixed to 1.5 TeV and we vary the dark matter mass $m_X$ from 200 to 900 GeV.

We first consider scenarios with couplings satisfying Eqs. (9) and (10). We evaluate fiducial NLO cross sections for the different considered mass spectra (second and fifth columns of the table for the **S1** and **S2** cases respectively), after imposing the previously-mentioned cuts on the transverse momentum of the leading jet $p_T(j_1) > 150$ GeV and on the parton-level missing transverse energy  GeV. For both the **S1** and **S2** scenarios, the NLO predictions are found to decrease with the dark matter mass, paying the price of a phase-space suppression. The falling behaviour is found steeper once the dark matter mass is greater than half the mediator mass, as it has to be produced off-shell (*i.e.* for $m_Y>2m_X$). Moreover, for given masses and couplings, **S1** cross sections (*i.e.* in the case of mediator vector couplings) are larger. This originates from the *p*-wave suppression of DM production through an axial-vector mediator (*i.e.* in the **S2** scenario), as already mentioned earlier in this section.

We then evaluate the cross section value $\sigma _{95}$ that is excluded at the 95% confidence level (third and sixth columns of the table) by reinterpreting the results of the ATLAS-CONF-2019-040 analysis. We observe that small dark matter masses are excluded already with the full Run 2 dataset, cross sections as small as 100 fb being excluded regardless of the DM mass. Moving on with a scenario in which the couplings satisfy Eqs. (9) and (11), we translate the bounds that we have obtained into bounds on a universal coupling. The latter is found to be of at most in the 0.5–0.7 range once one lies in a configuration below threshold ($2 m_X < m_Y$), and is mostly unconstrained for larger DM mass values. As for the previous class of scenarios in which the DM mass was fixed and the mediator mass was varying, 3000 fb$^{-1}$ will not improve the limits much, as the analysis being dominated by the systematics. We indeed expect an improvement on the bounds of at most 3–4%.

## Conclusion

In this paper we showcased new features of the MadAnalysis 5 package that improve the recasting functionalities of the programme. These features focus on two aspects.

First, we have designed a way to include the uncertainties on the signal when the code is used to reinterpret given LHC results in different theoretical contexts. Theory errors on the total signal production cross section induced by scale and PDF variations can be propagated through the reinterpretation procedure. This results in an uncertainty band attached to the confidence level at which a given signal is excluded. In addition, the user has the option to provide information on the systematic uncertainties on the signal. With the existence of new physics masses being pushed to higher and higher scales, keeping track of error information on the signal becomes mandatory, especially for what concerns the theoretical uncertainties, which can be significant for beyond the Standard Model physics signals involving heavy particles.

Second, we have implemented a new option allowing the user to extrapolate the constraining power of any specific analysis to a different luminosity, assuming a naive scaling of the signal and background selection efficiencies. Several options are available for the treatment of the background uncertainties, depending on the information provided by the experimental collaborations for the analyses under consideration. If information on the statistical and systematical components of the uncertainties is available separately, signal region by signal region, MadAnalysis 5 can use it to scale them up accordingly, *i.e.* proportionally to the square root of the luminosity and linearly to the luminosity for the statistical and systematical uncertainties respectively. In contrast, if such a detailed information is absent, the user is offered the choice to treat the total error as being dominated either by statistics or by systematics, or in his/her preferred fashion.

We have illustrated the usage of these new MadAnalysis 5 features in the framework of three simplified models for new physics.

First, we consider a signal that originates from the production of a pair of gluinos that each decay into two jets and missing transverse momentum. As an example, we make predictions in the context of a simplified model inspired by the MSSM, in which only the gluino and the lightest neutralino are light enough to be reachable at the LHC. We have investigated the potential of two ATLAS searches for supersymmetry in 36 fb$^{-1}$ and 139 fb$^{-1}$ of LHC data. Those searches both rely on the effective mass variable and on the presence of a large amount of missing transverse energy, and include a large variety of signal regions featuring different jet multiplicity and hadronic activity. We have reproduced to a good approximation the ATLAS results at the nominal luminosity of the analysis and compared our extrapolations at higher luminosities with those obtained either through the more naive approach of the Collider Reach platform, or to publicly available ATLAS estimates for the high-luminosity runs of the LHC. Fair agreement has been found. We have moreover studied the differences in the expected sensitivity that arise when one considers, as a starting point, an analysis of 36 or 139 fb$^{-1}$ of Run 2 LHC data. Our predictions are found fairly compatible, once theory errors are accounted for.

Second, we have focused on another MSSM-inspired simplified model in which the SM field content is supplemented by one species of first generation squark and one neutralino, all other supersymmetric states being decoupled. The spectrum configuration is therefore such that the squark is heavier than the neutralino and thus always decay into a light quark and a neutralino. This gives rise to a multijet plus missing transverse energy signatures stemming from squark pair production and decay. However, in contrast with the gluino case, a smaller signal jet multiplicity is expected. We have considered the same ATLAS supersymmetry search in 139 fb$^{-1}$ of LHC data as above-mentioned, as it includes signal regions with a smaller jet multiplicity so that some sensitivity to the considered simplified model is expected. We have reinterpreted the results of the search and derived up-to-date constraints on the model. We have then extrapolated our findings to the high-luminosity LHC case.

Finally, we have considered an *s*-channel dark matter simplified model in which one extends the Standard Model by a single dark matter candidate and one mediator that connects the dark sector (made of the dark matter state) to the SM sector. We have considered a fermionic dark matter state and a spin-1 mediator that couples to a pair of SM quarks and a pair of dark matter particles. Typical dark matter signals hence arise from the production of a pair of dark matter particles (through an *s*-channel mediator exchange) in association with a hard visible object. The most common case involves the production of a jet with a pair of invisible dark matter particles, the signal being dubbed monojet in this case. As the above ATLAS search is sensitive to such a signature (by virtue of the properties of its low jet multiplicity signal regions), we reinterpret its results to constrain the simplified model under consideration. We focus on and compare two cases where the mediator couplings are of a vector and axial-vector nature respectively. We then extract the current limits on the model, and additionally project them at a higher-luminosity to get estimates for the LHC sensitivity to the two studied *s*-channel dark matter setups.

In all the models investigated , our results emphasise the importance of embedding the uncertainties on the signal. In one considered example, this could degrade the expected bounds by about 10–20%, especially as a consequence of the large theory errors originating from the poor PDF fit constraints at large Bjorken-*x*. Such a regime is indeed relevant for new physics configurations still allowed by current data and that involve the production of massive particles lying in the multi-TeV mass range.

## Data Availability

This manuscript has no associated data or the data will not be deposited. [Authors comment: This is a theory work, so that there is no data.].

## Footnotes

1 See the webpage https://madanalysis.irmp.ucl.ac.be/wiki/PublicAnalysisDatabase.

2 See the webpage https://launchpad.net/madanalysis5.

3 See the webpage https://root.cern.ch.

4 See the webpage https://twiki.cern.ch/LHCPhysics/SUSYCrossSections13TeVgluglu.

5 See the webpage http://collider-reach.web.cern.ch.

6 See the webpage http://feynrules.irmp.ucl.ac.be/wiki/DMsimp.
